# L-plastin Ser5 phosphorylation is modulated by the PI3K/SGK pathway and promotes breast cancer cell invasiveness

**DOI:** 10.1186/s12964-021-00710-5

**Published:** 2021-02-22

**Authors:** Raquel A. C. Machado, Dunja Stojevski, Sébastien De Landtsheer, Philippe Lucarelli, Alexandre Baron, Thomas Sauter, Elisabeth Schaffner-Reckinger

**Affiliations:** 1 Cancer Cell Biology and Drug Discovery Group, Department of Life Sciences and Medicine, Faculty of Science, Technology and Medicine, University of Luxembourg, CAMPUS Belval, BT1, 7, avenue des Hauts-Fourneaux, 4362 Esch-sur-Alzette, Luxembourg; 2 Systems Biology Group, Department of Life Sciences and Medicine, University of Luxembourg, Esch-sur-Alzette, Luxembourg; 3 Present Address: Sensile Medical AG, Olten, Switzerland; 4 Present Address: Immune Systems Biology Group, Department of Infection and Immunity, Luxembourg Institute of Health, Esch-sur-Alzette, Luxembourg

**Keywords:** L-plastin, Actin-bundling, PI3K pathway, ERK/MAPK pathway, SGK, RSK, Invasion, Invadopodia, Extracellular matrix degradation, Metastasis

## Abstract

**Background:**

Metastasis is the predominant cause for cancer morbidity and mortality accounting for approximatively 90% of cancer deaths. The actin-bundling protein L-plastin has been proposed as a metastatic marker and phosphorylation on its residue Ser5 is known to increase its actin-bundling activity. We recently showed that activation of the ERK/MAPK signalling pathway leads to L-plastin Ser5 phosphorylation and that the downstream kinases RSK1 and RSK2 are able to directly phosphorylate Ser5. Here we investigate the involvement of the PI3K pathway in L-plastin Ser5 phosphorylation and the functional effect of this phosphorylation event in breast cancer cells.

**Methods:**

To unravel the signal transduction network upstream of L-plastin Ser5 phosphorylation, we performed computational modelling based on immunoblot analysis data, followed by experimental validation through inhibition/overexpression studies and in vitro kinase assays. To assess the functional impact of L-plastin expression/Ser5 phosphorylation in breast cancer cells, we either silenced L-plastin in cell lines initially expressing endogenous L-plastin or neoexpressed L-plastin wild type and phosphovariants in cell lines devoid of endogenous L-plastin. The established cell lines were used for cell biology experiments and confocal microscopy analysis.

**Results:**

Our modelling approach revealed that, in addition to the ERK/MAPK pathway and depending on the cellular context, the PI3K pathway contributes to L-plastin Ser5 phosphorylation through its downstream kinase SGK3. The results of the transwell invasion/migration assays showed that shRNA-mediated knockdown of L-plastin in BT-20 or HCC38 cells significantly reduced cell invasion, whereas stable expression of the phosphomimetic L-plastin Ser5Glu variant led to increased migration and invasion of BT-549 and MDA-MB-231 cells. Finally, confocal image analysis combined with zymography experiments and gelatin degradation assays provided evidence that L-plastin Ser5 phosphorylation promotes L-plastin recruitment to invadopodia, MMP-9 activity and concomitant extracellular matrix degradation.

**Conclusion:**

Altogether, our results demonstrate that L-plastin Ser5 phosphorylation increases breast cancer cell invasiveness. Being a downstream molecule of both ERK/MAPK and PI3K/SGK pathways, L-plastin is proposed here as a potential target for therapeutic approaches that are aimed at blocking dysregulated signalling outcome of both pathways and, thus, at impairing cancer cell invasion and metastasis formation.

**Video abstract**

## Background

Cells respond to intra- and extracellular changes by triggering intracellular signalling events, which are necessary for eliciting and regulating normal cell processes. Aberrant signalling can lead to disease [1] and cancer is commonly considered as a cell signalling disorder [2]. In particular, the ERK/MAPK and the PI3K pathways are two of the most commonly dysregulated signal transduction pathways in breast cancer [3] and harbour cancer driver genes that are associated with many cancer types [4]. Among other stimuli, both pathways are activated in response to growth factor binding to their respective receptors endowed with tyrosine kinase activity (RTKs) and their role in the control of cell proliferation, survival, differentiation, metabolism and invasion/migration has long been established [5]. Increased signalling often occurs as a result of mutation and/or amplification of key components of the pathways, which commonly lead to hyper-activation of downstream effectors. Moreover, there exists an extensive crosstalk between the two pathways and inhibition of one pathway may be compensated by activation of the other pathway, thus leading to attenuation of targeted therapeutic efficacy and contributing to drug resistance [6].

The actin-bundling protein L-plastin or fimbrin is encoded by the LCP1 gene and has been initially detected in leukocytes where it plays a role in the immune response [7]. In addition, L-plastin is often ectopically expressed in malignant cells of non-hematopoietic origin [8, 9]. As both leukocytes and cancer cells are capable of moving, the expression of L-plastin seems to be characteristic for motile cells and the involvement of L-plastin in immune cell motility is well established [10]. In the cancer context, our group has previously described that L-plastin is highly enriched in actin-based structures playing a role in cell migration, such as ruffling membranes, microspikes, and filopodia-like structures [11–13]. Notably, L-plastin also localizes in structures involved in cell invasion such as podosomes in cells of the immune system and invadopodia in cancer cells. Indeed, L-plastin localization to podosomes has been reported in macrophages [14, 15], in monocyte-derived osteoclasts [16, 17] and in neutrophils [18]. Moreover, a recent report has provided evidence for the recruitment of L-plastin to invadopodia [19], which are structures that mediate dissemination and metastasis of cancer cells [20]. Importantly, phosphorylation of L-plastin on its residue serine-5 (Ser5) contributes to protein activation and increases its actin-bundling activity [11, 12]. In this regard, several studies have shown that not only L-plastin expression, but mostly L-plastin phosphorylation should be considered when linking L-plastin to tumor progression [21]. Therefore, the elucidation of the signalling network upstream of L-plastin Ser5 phosphorylation is of high interest.

In our recent work, we have shown that ERK/MAPK signalling leads to Ser5 phosphorylation of L-plastin and that this phosphorylation can be directly mediated by the ERK/MAPK pathway downstream kinases RSK1 and RSK2 [13]. Notably, our in vitro kinase screening assays pointed to a potential role of additional kinases including SGKs and p70S6K, all of which are located downstream of PI3K. Since the PI3K pathway is also commonly dysregulated in breast cancer [3] and given the existence of an extensive crosstalk between the ERK/MAPK and PI3K pathways in addition to their prominent role in a large plethora of cancer-associated processes [6], we sought to extend our investigations and assess the involvement of the PI3K pathway in L-plastin Ser5 phosphorylation in breast cancer cells. Taking a computational modelling approach, followed by experimental validation, we found that the PI3K pathway, in addition to the ERK/MAPK pathway, plays a role in L-plastin Ser5 phosphorylation predominantly through its downstream kinase SGK3. Of note, the contribution of the PI3K/SGK3 axis to L-plastin Ser5 phosphorylation strongly depends on the cellular context.

In order to explore the functional outcome of L-plastin expression and, in particular, of L-plastin Ser5 phosphorylation, we have investigated their role in invasion/migration processes, invadopodia formation and extracellular matrix (ECM) degradation. To this end, we have taken two parallel approaches: first, we have silenced L-plastin expression in two breast cancer cell lines initially expressing endogenous L-plastin and, second, we have neoexpressed L-plastin wild type or the phosphorylation variants L-plastin Ser5Ala (S5A, non-phosphorylatable) or L-plastin Ser5Glu (S5E, phosphomimetic) in breast cancer cells expressing only a low level or no endogenous L-plastin. Our results indicate that L-plastin expression and especially L-plastin Ser5 phosphorylation enhances invasion/migration of breast cancer cells. Furthermore, Ser5 phosphorylation increases the recruitment of L-plastin to invadopodia as well as ECM degradation.

## Methods

### Cell culture, cell transfection and generation of stable cell clones

MCF7 (# HTB-22, RRID:CVCL_0031), HCC38 (# CRL-2314, RRID:CVCL_1267), BT-549 (# HTB-122, RRID:CVCL_1092) and MDA-MB-231 (# HTB-26, RRID:CVCL_0062) cells were cultured in Roswell Park Memorial Institute medium, SKBR3 (# HTB-30, RRID:CVCL_0033) cells in McCoy’s 5A medium, BT-20 (# CRL-7912, RRID:CVCL_0178) cells in Eagle’s minimal essential medium and HEK 293T (# CRL-3216, RRID:CVCL_0063) cells in Dulbecco’s modified Eagle’s medium (Lonza Group, Basel, Switzerland). All media were supplemented with 10% fetal bovine serum and 2 mM L-glutamine (complete medium) (Lonza Group). Cells were grown at 37 °C in a H_2_O-saturated, 5% CO_2_ atmosphere. Cells were either bought from or authenticated by American Type Culture Collection (Manassas, VA, USA).

Co-transfection of HEK 293T cells with plasmids encoding FLAG-tagged SGK3 constructs and pEGFP-N1 L-plastinWT was performed using Lipofectamine 2000 following the manufacturer’s instructions (Invitrogen, Carlsbad, CA, USA). Cells were harvested 48 h after transfection and used for immunoblot analysis.

For immunoprecipitation experiments, HEK 293T cells were transfected with the plasmids pEGFP-N1 L-plastinWT, pEGFP-N1 L-plastinS5E, pEGFP-N1 L-plastinS5A or pEGFP-N1 L-plastinEF-ABD1 using Lipofectamine 2000 (Invitrogen). Cells were harvested 24 h after transfection and used for immunoprecipitation.

HEK 293T cells were used for the production of lentiviral particles. Briefly, HEK 293T cells were transiently transfected with third generation lentiviral vectors using Lipofectamine 2000. The virus-containing supernatant was harvested 24 h and 48 h after medium change, cleared by centrifugation at 2000 rpm and 4 °C for 10 min, and filtered through a 0.45 μm filter. Concentration of lentiviral particles was performed by precipitation with PEG10000 (1:5 volume of 40% PEG10000 solution; Merck KGaA, Darmstadt, Germany) at 4 °C overnight, followed by centrifugation at 2800 rpm and 4 °C for 30 min. The virus pellet was resuspended in serum-free medium, divided in aliquots, and stored at − 80 °C. Target cells were transduced in the presence of 8 μg/ml Polybrene (hexadimethrine bromide, Merck) for 16 h. The transduced cells, positive for green fluorescent protein (GFP) expression, were selected with 1 μg/ml puromycin in complete medium for 48 h.

### Plasmids

The plasmid pEGFP-N1 L-plastinWT used for transiently transfecting HEK 293T cells was generated from the previously described plasmid pDsRed-Monomer-N1 L-plastinWT [12]. Briefly, the L-plastinWT 1.9 kb cDNA fragment obtained by *Eco*RI/*Age*I restriction of pDsRed-Monomer-N1 L-plastinWT was inserted into the *Eco*RI/*Age*I restricted pEGFP-N1 vector. The plasmid pEGFP-N1 L-plastinEF-ABD1 was generated by PCR amplification using the plasmid pEGFP-N1 L-plastinWT as a template and using primers that were designed to generate the restriction sites *Eco*RI and *Bam*HI necessary for cloning the PCR-amplified cDNA into the pEGFP-N1 vector. The following primers were used: 5′-TATAGAATTCATGGCCAGAGGATC-3′ as forward primer and 5′-GCGGATCCGCTTTGTGCAGGGC-3′ as reverse complement primer. Lentiviral transduction was performed using third generation lentiviral vectors. The packaging vector psPAX2 and the envelope vector pMD2.G were obtained from Addgene (LGC Standards, Middlesex, United Kingdom). The transfer vector CD527A-1 carried the cDNAs corresponding to GFP, L-plastinWT-GFP, non-phosphorylatable L-plastinS5A-GFP or phosphomimetic L-plastinS5E-GFP. Briefly, the cDNA fragments were obtained by PCR amplification using the respective pEGFP-N1 plasmids as templates and using primers that were designed to generate the requested L-plastin mutation as well as the restriction sites necessary for cloning the PCR-amplified cDNAs into the CD527A-1 vector. For all cDNAs, *Xba*I and *Bam*HI restriction sites were generated at the 5′- and 3′-ends, respectively. The following primers were used: 5′-TACTTCTAGAATGGCCAGAGGATCAGTGTC-3′ as forward primer for L-plastinWT-GFP, 5′-TACTTCTAGAATGGCCAGAGGAGCAGT-3′ as forward primer for L-plastinS5A-GFP, 5′-TACTTCTAGAATGGCCAGAGGAGAAGTGTC-3′ as forward primer for L-plastinS5E-GFP, 5′-TACTTCTAGAATGGTGAGCAAGGGCGA-3′ as forward primer for GFP and finally 5′-AGTAGGATCCCTTGTACAGCTCGTCCATGC-3′ as reverse complement primer for all constructs. All constructs were verified by sequencing. The GIPZ short hairpin RNA (shRNA) non-silencing lentiviral vector as well as the target shRNAs for L-plastin (GIPZ Lentiviral shRNA Library, pool of clones V2LHS_133928, V2LHS_133929, V2LHS_238253, V2LHS_311716 and V2LHS_311717) were purchased from GE Dharmacon (Diegem, Belgium). The FLAG-tagged SGK3 plasmids were a kind gift of Professor Dan Liu (Baylor College of Medicine, Houston, TX, USA). Notably, the myristoylated SGK3 construct was obtained by adding the N-terminal myristoylation sequence of chicken c-Src to the 5′-end of SGK3 (Myr SGK3) (characterized in [22]).

### Antibodies and reagents

Antibodies mouse anti-Src (L4A1, #2110, RRID:AB_10691385), rabbit anti-EGFR (#2232, RRID:AB_331707), rabbit anti-IGF-IRβ (D23H3, #9750, RRID:AB_10950969), rabbit anti-phosphoSrc (pTyr416, #2101, RRID:AB_331697), rabbit anti-phosphoAKT (pSer473, D9E, #4060, RRID:AB_2315049) and rabbit anti-phosphoSGK3 (pThr320, D30E6, #5642, RRID:AB_10694357) were from Cell Signaling Technology (Danvers, MA, USA), goat anti-AKT (N-19, #sc-1619, RRID:AB_671713), mouse anti-phosphoERK (pTyr204, E-4, #sc-7383, RRID:AB_627545), mouse anti-SGK3 (C-6, #sc-166847, RRID:AB_2188556) and rabbit anti-cortactin (H-191, #sc-11408, RRID:AB_2088281) from Santa Cruz Biotechnology, Inc. (Dallas, TX, USA), rabbit anti-ERK (#M5670, RRID:AB_477216), mouse anti-cortactin (4F11, #05-180, RRID:AB_309647) and mouse anti-β-actin (#A5441, RRID:AB_476744) from Merck, rabbit anti-HGFR (22H22L13, #700261, RRID:AB_2532310) and mouse anti-L-plastin (LPL4A.1, #MA5-11921, RRID:AB_10979969) from Thermo Fisher Scientific (Waltham, MA, USA), mouse anti-MDM2 (2A10, #ab16895, RRID:AB_2143534) and rabbit anti-phosphoMDM2 (pSer166, #ab170880,) from Abcam (Cambridge, United Kingdom). The GFP-Trap_A antibody used for the nanotrap assays (#gta-20, RRID:AB_2631357) was from Chromotek (Planegg, Germany). The rabbit antibody specifically recognizing L-plastin phosphorylated on Ser5 (anti-Ser5-P) was raised against a peptide encoding L-plastin residues 2–17 where Ser5 was phosphorylated [ARGS(P)VSDEEMMELREA] (characterized in [11]).

The stimulators phorbol 12-myristate 13-acetate (PMA), human epidermal growth factor (EGF), human hepatocyte growth factor (HGF) and human insulin-like growth factor 1 (IGF) were purchased at Merck. The inhibitors FAK inhibitor II and RSK inhibitor II (Bi-D1870) were from Merck and AKT inhibitor VIII was from VWR (Oud-Heverlee, Belgium). The MEK inhibitor Trametinib and the dual PI3K/mTOR inhibitor Apitolisib were purchased at CliniSciences (Nanterre, France). Recombinant human full-length MDM2 protein was obtained from Abcam. Human tissue microarrays (TMAs) were purchased at AMS Biotechnology (Cambridge, MA, USA).

### Treatment of cells with pharmacologic agents

Cells were cultured in the absence of serum for 16 h and then treatment was performed at 37 °C as follows: 0.1 µM PMA for 1 h, 1 ng/ml EGF for 15 min, 40 ng/ml HGF for 20 min, 100 ng/ml IGF for 20 min, 20 µM AKT inhibitor VIII for 1 h, 5 µM FAK inhibitor II for 1 h, 5 µM RSK inhibitor Bi-D1870 for 30 min, 5 nM Trametinib (Mekinist) for 1 h or 500 nM Apitolisib for 1 h. When activators and inhibitors were combined, the incubation with the inhibitors was performed first and their presence was maintained during the incubation with the activators.

### Immunoblot analysis

In situ cell lysis was performed with a cell scraper in ice-cold lysis buffer (50 mM Tris–HCl pH 7.5, 150 mM NaCl, 0.1% SDS, 5 mM EDTA, 1% Nonidet P-40, 1% Triton X-100, 1% sodium-deoxycholate, 1 mM Na_3_VO_4_, 10 mM NaF, 100 mM leupeptin, and 100 mM E64D) containing a cocktail of protease inhibitors (Roche Diagnostics GmbH, Mannheim, Germany). Lysate clarification was done by centrifugation at 13,200 rpm for 15 min at 4 °C and total protein concentration was determined by Bradford assay (Bio-Rad, Hercules, CA, USA). Proteins (50 µg per lane) were resolved by SDS-PAGE in a 10% NuPAGE Tris-Base gel (Invitrogen) under reducing conditions, and transferred to a nitrocellulose membrane (GE Healthcare, Chicago, IL, USA) by semidry transfer. Membranes were saturated in Tris-buffered saline containing 1% bovine serum albumin and 0.1% Tween for 1 h at room temperature, then incubated with primary antibodies overnight at 4 °C and with secondary antibodies IRDye 680 RD donkey anti-mouse (#926–68,072, RRID:AB_10953628, Thermo Fisher Scientific) and IRDye 800 CW goat anti-rabbit (#926–32,211, RRID:AB_621843, Thermo Fisher Scientific) for 1 h at room temperature. Each antibody incubation was followed by at least three wash steps in Tris-buffered saline supplemented with 0.1% Tween. Signal intensities were quantified using the Odyssey Infrared Image System (LI-COR Biosciences, Lincoln, NE, USA). The ratio between the intensities obtained for phosphorylated protein versus total protein was calculated to make individual samples comparable and then normalized to the mean of all the ratios calculated for one blot to make blots comparable by accounting for technical day-to-day variability. For representative purposes, data were scaled to the controls present on each blot and are represented as means ± SEM of three independent experiments. Raw images of the immunoblots are shown in the Additional file 1: Figure S1.

### Modelling

The candidate signalling network upstream of L-plastin was derived from the literature as follows. The Ras/MAPK and PI3K/AKT pathways as well as cross-talk and compensation of the two pathways were derived from [5, 23, 24]. SGK, FAK, Src, PKA and PKC were integrated in the obtained network mainly based on [13, 25–32]. The experimental data were obtained by immunoblot analysis as described above and the ratios of P-LPL/LPL, P-ERK/ERK, P-AKT/AKT and P-Src/Src were used for model contextualization as follows. Within the FALCON toolbox, Dynamic Bayesian Networks are used to quantitatively simulate the logic of signalling pathways [33]. Briefly, networks are initialized in a random state and the activity of ‘input nodes’ is fixed according to the experimental conditions (presence or absence of growth factors and inhibitors). The signals are then propagated according to the laws of probability until convergence, when the activities of the ‘output nodes’ are compared with the measurements. A gradient descent algorithm is used to optimize the weights of the edges controlling the relative contributions of upstream nodes to downstream nodes in order to minimize the mean squared error (MSE) between the simulations and the measurements.

Regularized optimization was then used to put in evidence the specific differences in signalling between the cell lines. Two types of regularization were applied to the parameter space during joint optimization of the individual cell line-specific models. Firstly, we sought to decrease the influence of experimental noise on the results by including a group partial-norm term penalizing the concurrent activation of a node by more than one activator. The effect of such regularization is to prune the network of edges that are not well supported by experimental evidence. Secondly, uniformity regularization [34] was applied across the four cell lines for each parameter. This density-based regularization term stems from the biological assumption that differences between the cell lines are more likely due to a small number of differences than to large-scale rewiring, and its effect is to remove small differences between cell line-specific models unless they are well supported by the data. The combined effect of these two regularization terms is to reduce the size of the model and point to the signalling pathways that are differentially activated among the cell lines.

Regularized optimization with the FALCON toolbox was performed on the full dataset, after which the optimal model size was determined using the Bayesian Information Criterion [35] and the topology of the final multi-cell line model was fixed by removing edges with low (< 0.01) flux and merging similar (< 0.01 standard deviation) edges. This final model was re-optimized on the full dataset, using unregularized optimization, to retrieve unbiased estimations for the activity of the different signalling proteins and the strength of the interactions between them. To estimate the error on the parameters, we optimized 20 models with synthetic datasets by applying random Gaussian noise on the measurements proportionally to the measurement error. Files containing the data used for the modelling can be found in the Additional file 2: Figure S4, Additional file 3: Figure S5, Additional file 4: Figures S6, Additional file 5: Figure S7, Additional file 6: Figure S8, Additional file 7: Figure S9, Additional file 8: Figure S10, Additional file 9: Figure S11, Additional file 10: Figure S12, Additional file 11: Figure S13, Additional file 12: Figure S14.

### In vitro kinase assays of full-length recombinant L-plastin

The in vitro kinase assay was carried out as described before (Lommel, 2016). Briefly, full-length recombinant L-plastin (10 µg) or full-length recombinant MDM2 (2 µg) were incubated with 50 µM ATP and 100 ng recombinant kinase SGK1, SGK2, SGK3 or RSK1 purchased at SignalChem (Richmond, BC, Canada) in a reaction volume of 25 µl, according to the manufacturer’s protocol. For the negative control, the respective kinase was omitted. For full-length L-plastin, a positive control reaction was performed using the kinase RSK1. Following an incubation of 1 h at 30 °C, Laemmli buffer was added, and the samples were boiled at 100 °C for 5 min and then subjected to immunoblot analysis.

### Immunoprecipitation

For immunoprecipitation, 6 × 10^6^ HEK 293 T cells were transiently transfected with expression vectors encoding GFP, L-plastinWT-GFP, L-plastinS5A-GFP, L-plastinS5E-GFP or L-plastinEF-ABD1-GFP. 24 h after transfection, cells were homogenized in 500 µl lysis buffer (50 mM Tris–HCl pH 7.5, 150 mM NaCl, 0.5 mM EDTA, 1% Triton, 1% glycerin, 1 mM PMSF, 1 mM sodium orthovanadate) containing a cocktail of protease inhibitors (Roche Diagnostics) and incubated on ice for 30 min. After a centrifugation step at 13,200 rpm and 4 °C for 10 min, total protein concentration was determined by Bradford assay (Bio-Rad) and sample concentrations were adjusted with dilution buffer (10 mM Tris–HCl pH 7.5, 150 mM NaCl, 0.5 mM EDTA, 1 mM PMSF, cocktail of protease inhibitors). 50 µl were added to SDS-containing sample buffer and used for SDS-PAGE (referred to as input). 25 µl of GFP-nanotrap beads (#gta-20, RRID:AB_2631357, Chromotek) were added and incubated for 2 h on an end-over-end rotor at 4 °C. After a centrifugation step of 5 min at 3000 rpm at 4 °C, the supernatant was removed, and 50 µl of the supernatant were used for SDS-PAGE (referred to as non-bound). The bead pellet was washed four times with 300 µl dilution buffer. After the last washing step, the beads were resuspended in 2 × SDS-containing sample buffer and boiled for 10 min at 95 °C (referred to as bound). The obtained samples were submitted to immunoblot analysis.

### Transwell migration and invasion assays

For the transwell assays, cells were washed in phosphate-buffered saline (PBS) and resuspended in serum-free medium. A cell suspension containing 50,000 cells was added to the upper well of transwell migration inserts (pore size: 8 μm, BD Biosciences, San Jose, CA, USA) or 100,000 cells to BD BioCoat™ Matrigel™ invasion chambers (pore size: 8 μm, BD Biosciences). In the lower well, complete medium (700 μl) was used as chemoattractant. Cells were incubated for 24 h at 37 °C and 5% CO2, fixed in 4% PFA for 10 min and stained with DAPI for 10 min. Cells that did not migrate to the lower compartment were removed with a cotton swab. Inserts were mounted on glass slides and five random fields at a magnification of 20 × were counted per sample.

### Immunofluorescence

Cells were plated on 0.1% gelatin-coated glass coverslips. Following incubation, cells were washed with PHEM buffer (2 mM HEPES, 10 mM EGTA, 2 mM MgCl_2_, 60 mM PIPES, pH 6.9) and fixed for 20 min with cold PFA 4%. Next, cells were permeabilized with 0.1% Triton X-100 for 10 min, blocked with 1% bovine serum albumin in PHEM buffer for 30 min, and then incubated with mouse anti-cortactin (1:200, #05–180, RRID:AB_309647, Merck) and rabbit anti-Ser5-P-L-plastin (1:50) at 4 ºC overnight, followed by incubation with Alexa Fluor 405-conjugated goat anti-mouse IgG (1:250, #A31553, RRID:AB_221604, Thermo Fisher Scientific), Alexa Fluor 488-conjugated GFP booster (1:200, #gb2AF488-10, RRID:AB_2827573, Chromotek), Alexa Fluor 594-conjugated goat anti-rabbit IgG (1:250, #A11037, RRID:AB_2534095, Thermo Fisher Scientific) and Alexa Fluor 633-conjugated phalloidin (1:50, #A22284, Thermo Fisher Scientific) or Alexa Fluor 568-conjugated phalloidin (1:50, #12380, Thermo Fisher Scientific) at room temperature for 1 h. Coverslips were mounted using Vectashield Anti-fade Mounting Medium (#H-1000, RRID:AB_2336789, Vector Laboratories, San Francisco, CA, USA) and image acquisition was performed using the Andor Spinning Disk Revolution system (CSU-W1; Andor Technology, Belfast, United Kingdom) based on a Nikon Ti microscope (Nikon, Tokyo, Japan) with an Andor iXon Ultra EMCCD camera.

### Invadopodia quantification

To quantify invadopodia formation, MDA-MB-231 cells were plated at low density on top of 0.1% gelatin-coated coverslips and cultured for 24 h. All samples from the same replicate were stained simultaneously as described above. Four random fields at a magnification of 40 × were counted per sample using single confocal slices of the ventral surface of the cells. Image analysis was performed using ImageJ software (RRID:SCR_003070, National Institutes of Health, Bethesda, MD, USA). Firstly, the threshold “moments” was applied to the images of cells stained for F-actin and cortactin. To identify invadopodia, the tool “image calculator” was used to show dot-like structures that were present in both images. The GFP-positive invadopodia were determined in the same way using the result image obtained from the calculation of F-actin and cortactin, which was then compared with the GFP signal. Particle frequency was determined using the “analyze particle” command. A cut-off of 0.5–20 µm^2^ was set as the size range and a value of 0.2 as the minimal circularity shape.

### Gelatin degradation assay

The gelatin degradation assay was adapted from a previously described protocol [36]. Firstly, 0.2% gelatin solution (#9000-70-8, Merck) was labeled using the Alexa Fluor 568-gelatin labeling kit (#A10238, Thermo Fisher Scientific) and aliquots were stored at − 20 °C. To coat glass coverslips, the fluorescent gelatin stock was mixed in a proportion 4:1 with non-labeled 0.2% gelatin solution and kept at 50 °C. A volume of 100 µl of this mixture was given on top of each coverslip and incubated for 5 min. The coverslips were lifted and submerged in PBS in separate wells in a 12-well cell culture plate. When all coverslips were coated, PBS was aspirated and coverslips were incubated for 15 min on ice with pre-chilled 0.5% glutaraldehyde solution. After washing, the coverslips were incubated for 3 min at room temperature with freshly prepared sodium borohydride solution (5 mg/ml). Finally, the coverslips were extensively washed and stored at 4 °C in PBS for up to two weeks, protected from light.

To quantify the gelatin degradation ability, 80,000 MDA-MB-231 cells were plated on top of Alexa Fluor 568-labeled gelatin-coated coverslips in 12-well cell culture plates and allowed to attach for 6 h. Coverslips were then submitted to immunofluorescence and six random fields at a magnification of 60 × were examined per sample using single confocal slices of the ventral surface of the cells. The cell area was determined by the F-actin staining, using the “ROI manager” tool of ImageJ software. To determine degraded area, a threshold was applied to make visible the dark areas of degraded fluorescent gelatin and quantification was performed using the “analyze particle” command. Relative degradation area was determined as total degradation area divided by total cell area, normalized to the value obtained for MDA-MB-231 GFP control cells.

### Zymography

To analyze the activity of matrix metalloproteinases (MMPs), cells were cultured in complete medium until 70–80% confluence. Cells were then washed with PBS and cultured in serum-free medium for 24 h. The conditioned medium was collected, cleared by centrifugation and stored at − 80 °C. Zymography acrylamide gels (10%) were prepared according to standard procedures with gelatin added to a final gelatin concentration of 1 mg/ml. The cell-free supernatant was mixed with 5 × non-reducing sample buffer, incubated at room temperature for 10 min, and a volume of 25 µl of the mixture was loaded on the gels. After electrophoresis, the gels were incubated in washing buffer (50 mM Tris–HCl pH 7.5, 5 mM CaCl_2_, 1 µM ZnCl_2_, 2.5% Triton X-100) for 30 min. Finally, the gels were kept at 37 °C with gentle agitation in incubation buffer (50 mM Tris–HCl pH 7.5, 5 mM CaCl_2_, 1 µM ZnCl_2_, 1% Triton X-100) for at least 24 h. Gelatinase activity was visualized by staining the gels with Coomassie Brilliant Blue G250 (Merck) with subsequent destaining in acetic acid–methanol–H_2_O (1:3:6). To visualize the amount of protein loaded, a parallel 10% polyacrylamide gel was loaded with the same volume of each sample and stained with Roti-blue (Carl Roth, Karlsruhe, Germany) for 1 h. Areas of protease activity and Roti-blue stained gels were analyzed using the Odyssey Infrared Image System (LI-COR Biosciences).

### Statistics

All statistical analyses were carried out using Prism 5 (GraphPad Software, RRID:SCR_002798, San Diego, CA, USA). Results are expressed as means ± SEM of three independent experiments. Statistical significance was assessed by performing unpaired Student’s t-test or ANOVA for multiple comparison tests.

## Results

### Analysis of growth factor-stimulated signalling in breast cancer cell lines

**Fig. 1:**
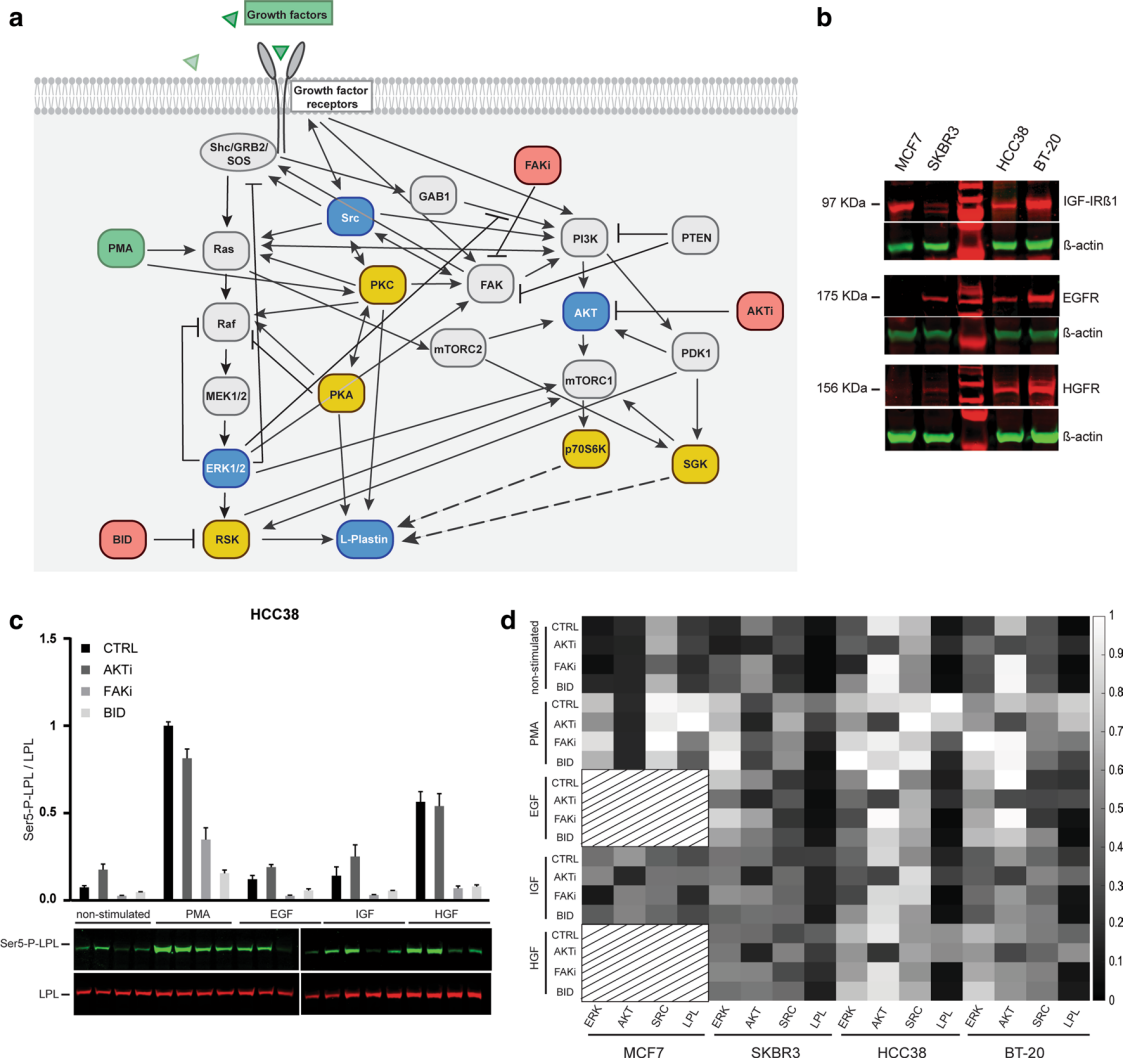
Literature-derived L-plastin signalling network and activation state of the output nodes. **a** A candidate network for the signalling pathways upstream of L-plastin Ser5 phosphorylation was built based on literature information. Green indicates the stimulators used, red the inhibitors, blue the output nodes and yellow the kinases upstream of L-plastin Ser5 phosphorylation. The dashed arrows represent the interactions that are under investigation. **b** Characterization of the investigated breast cancer cell lines. Determination of the expression of different growth factor receptors in MCF7, SKBR3, HCC38 and BT-20 cells. 50 µg of total cell extract were loaded per sample and β-actin was stained as a loading control. **c** Example of an immunoblot analysis for L-plastin Ser5 phosphorylation (Ser5-P-LPL) and total L-plastin (LPL). The graph shows the ratio between Ser5-P-L-plastin and L-plastin. The values are represented as means ± SEM of three independent experiments. **d** The heatmap represents the activation states (scaled between 0 and 1) of the four output nodes in four different cell lines for all the conditions tested

A candidate network of the regulatory signalling pathways upstream of L-plastin was assembled by manually curating signalling pathways from literature (Fig. 1a). Based on this network and in order to assess the interplay between ERK/MAPK and PI3K/AKT signalling pathways in regulating L-plastin Ser5 phosphorylation, we submitted four breast cancer cell lines to growth factor stimulation, with or without prior inhibition of key components of the two signalling pathways. Using MCF7, SKBR3, HCC38 and BT-20 cells, we first analyzed the expression level of different growth factor receptors. As verified by immunoblotting, the four cell lines express insulin-like growth factor 1 receptor (IGF-IR), although the level of expression is very low for SKBR3 cells. With the exception of MCF7 cells, the receptor for epidermal growth factor EGFR and the receptor for hepatocyte growth factor HGFR (or c-met) could be detected in all the investigated cell lines (Fig. 1b).

The RSK inhibitor II Bi-D1870 (BID) and the AKT inhibitor VIII were chosen to selectively block the ERK/MAPK and PI3K/AKT signalling pathways, respectively. As a more central player connecting both pathways, FAK was inhibited using FAK inhibitor II. Following treatment, cells were lysed and the ratio of the phosphorylation level versus the total protein level for different output nodes was determined as an indicator of their activation status. The investigated output nodes were ERK and AKT, which are commonly used as readouts for ERK/MAPK and PI3K/AKT signalling pathway activity, respectively. Additionally, we assessed the activation of the central player Src as well as L-plastin activation as the final output node. Growth factor stimulation was carried out based on the expression of the corresponding receptor by the respective cell line, as illustrated in Fig. 1b. In summary, we assessed the activation of four different output nodes in 20 different experimental conditions for the cell lines SKBR3, BT-20 and HCC38 and in 12 different experimental conditions for MCF7 cells. An example of the immunoblotting and the respective quantification of the ratios between Ser5-P-L-plastin and L-plastin for HCC38 cells is shown in Fig. 1c. The quantification of all output nodes activation in the four cell lines is shown in the Additional file 13: Figure S2 and these results are summarized as a heatmap indicating the activity level of the nodes in each condition (Fig. 1d).

### Modelling of the signalling network upstream of L-plastin

**Fig. 2:**
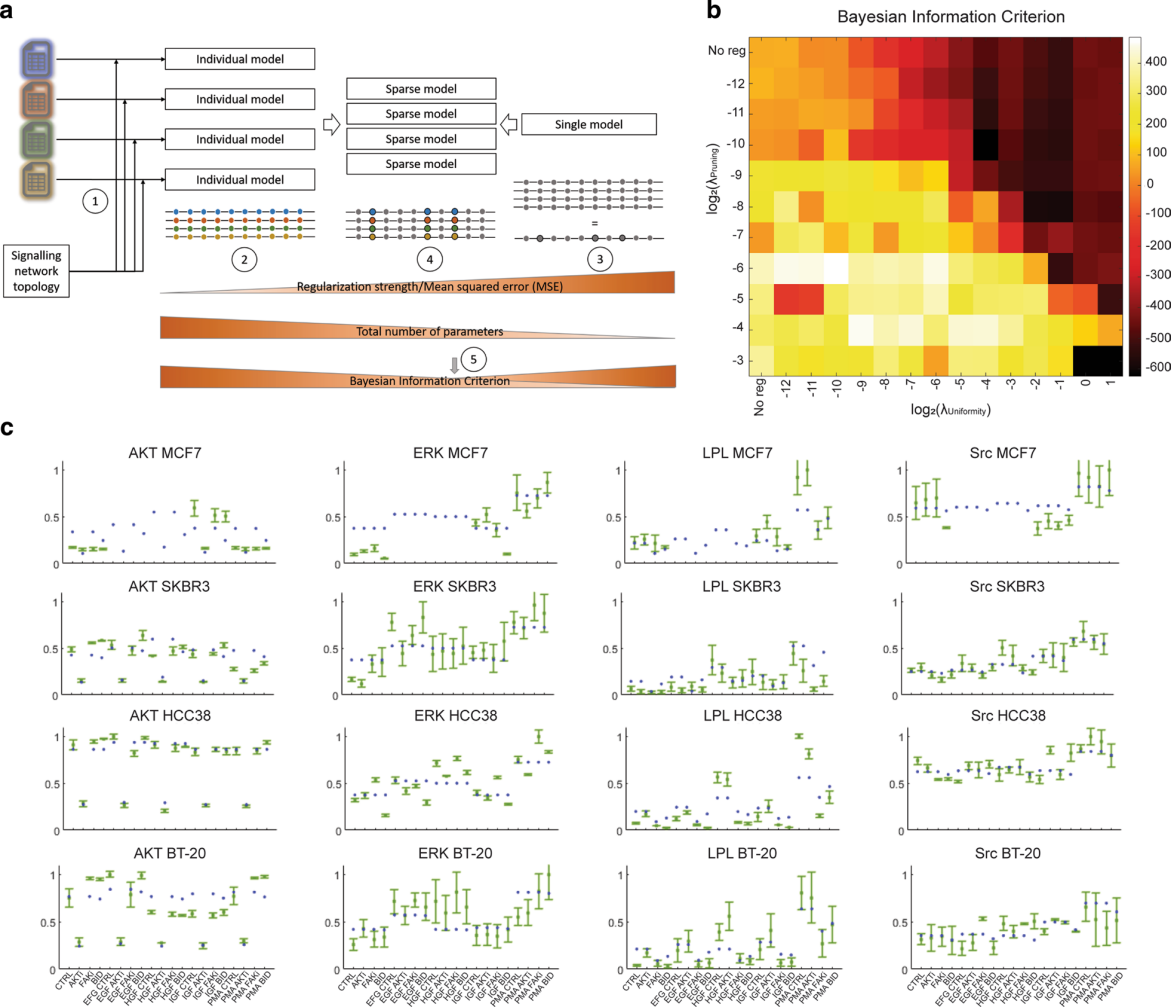
Computational modelling approach. **a** Inference of the cell line-specific parameters: (1) input data consist of a generic network topology and measurements of the activation state of output nodes (ratio phosphoprotein/total protein) for four cell lines, each cell line being represented by a different colour. (2) Without any regularization, model parameters are independent across cell lines, which might result in overfitting of the dataset. (3) When, in contrast, model parameters are forced to be equal across all cell lines, the phenotypes of the different cell lines are smoothed out and only the average behavior can be inferred. (4) By applying various levels of regularization (penalizing model size), the sparsity of the model, i.e. the number of model parameters allowed to vary across cell lines, can be controlled. (5) The Bayesian Information Criterion (BIC) is a measure of adequacy of the model, balancing model fitness and model size. The model configuration with the lowest BIC, evidencing the most crucial differences in signalling between the cell lines, was chosen for the final analyses. **b** Regularization landscape. Heatmap of the BIC values for each regularized model. Optimal model parametrization was obtained by screening values of the lambda_Pruning and lambda_Uniformity regularization parameters and computing the BIC for each resulting model. The model with the lowest BIC is considered the most adequate to represent the data and is obtained with log2(lambda_Pruning) = − 10 and log2(lambda_Uniformity) = − 4. **c** Best fit. Comparison of the simulated activity of the different measured proteins (output nodes) with the measurements. X-axis: different experimental conditions. Y-axis: normalized activity. Green: average of experimental measurements. The error bars represent 1 standard deviation. Blue: activity as simulated with the FALCON toolbox under the optimized final model topology

The averaged, normalized protein measurements were mapped to the corresponding network nodes, and the FALCON toolbox was then used to contextualize this network and retrieve, for each cell line specifically, the activity of the remaining nodes, the specific wiring of the signalling network and the flow of information for each experimental condition. Optimized regularization was performed to find the model that fits best the experimental data taking into account the cell-line specific parameters (Fig. 2a). The model with the lowest Bayesian Information Criterion (BIC) is considered the most adequate to represent the data (Fig. 2b), which corresponds to a model in which 63 of the 69 network parameters can be parametrized identically for all cell lines. Notably, interactions relating to the PI3K/AKT/mTOR axis showed relatively high heterogeneity compared to the crosstalks between them. The goodness-of-fit was similar for all cell lines (Fig. 2c), with MSE values ranging from 0.008 to 0.017 for individual cell lines, 0.032 for the single model and 0.018 for the final model. It should be noted that, in our final model, RSK, SGK, PKA and PKC appear as the kinases able to phosphorylate L-plastin on its residue Ser5, with RSK and SGK being the most prominent kinases (Table 1). Importantly, SGK as a downstream kinase of the PI3K pathway was pointed out as a novel kinase involved in this process.

**Table 1:** Optimized parameter values for the different models

	Single model Parameter value (± SD)	BT-20 model Parameter value (± SD)	HCC38 model Parameter value (± SD)	MCF7 model Parameter value (± SD)	SKBR3 model Parameter value (± SD)	Final model Parameter value (± SD)
p70S6K- > LPL	0.02 (± 0.03)	0.01 (± 0.04)	0.01 (± 0.01)	0.00 (± 0.02)	0.01 (± 0.06)	0.00 (± 0.00)
PKA- > LPL	0.02 (± 0.04)	0.01 (± 0.05)	0.08 (± 0.05)	0.13 (± 0.07)	0.08 (± 0.02)	0.13 (± 0.07)
PKC- > LPL	0.02 (± 0.04)	0.01 (± 0.05)	0.08 (± 0.05)	0.13 (± 0.07)	0.08 (± 0.02)	0.13 (± 0.07)
RSK- > LPL	0.50 (± 0.15)	0.72 (± 0.20)	0.79 (± 0.08)	0.74 (± 0.10)	0.35 (± 0.12)	0.28 (± 0.05)
SGK- > LPL	0.43 (± 0.14)	0.25 (± 0.16)	0.05 (± 0.09)	0.00 (± 0.18)	0.49 (± 0.16)	0.46 (± 0.14)

The optimized parameter values for the different models are expressed as the strength of the interaction from the parent node to the child node, relative to the total (= 1). Indicated is the value of the parameter for the best of all fits. The indicated error is the standard deviation (SD) of 20 rounds of re-sampling.

### PI3K is involved in the process of L-plastin Ser5 phosphorylation

**Fig. 3:**
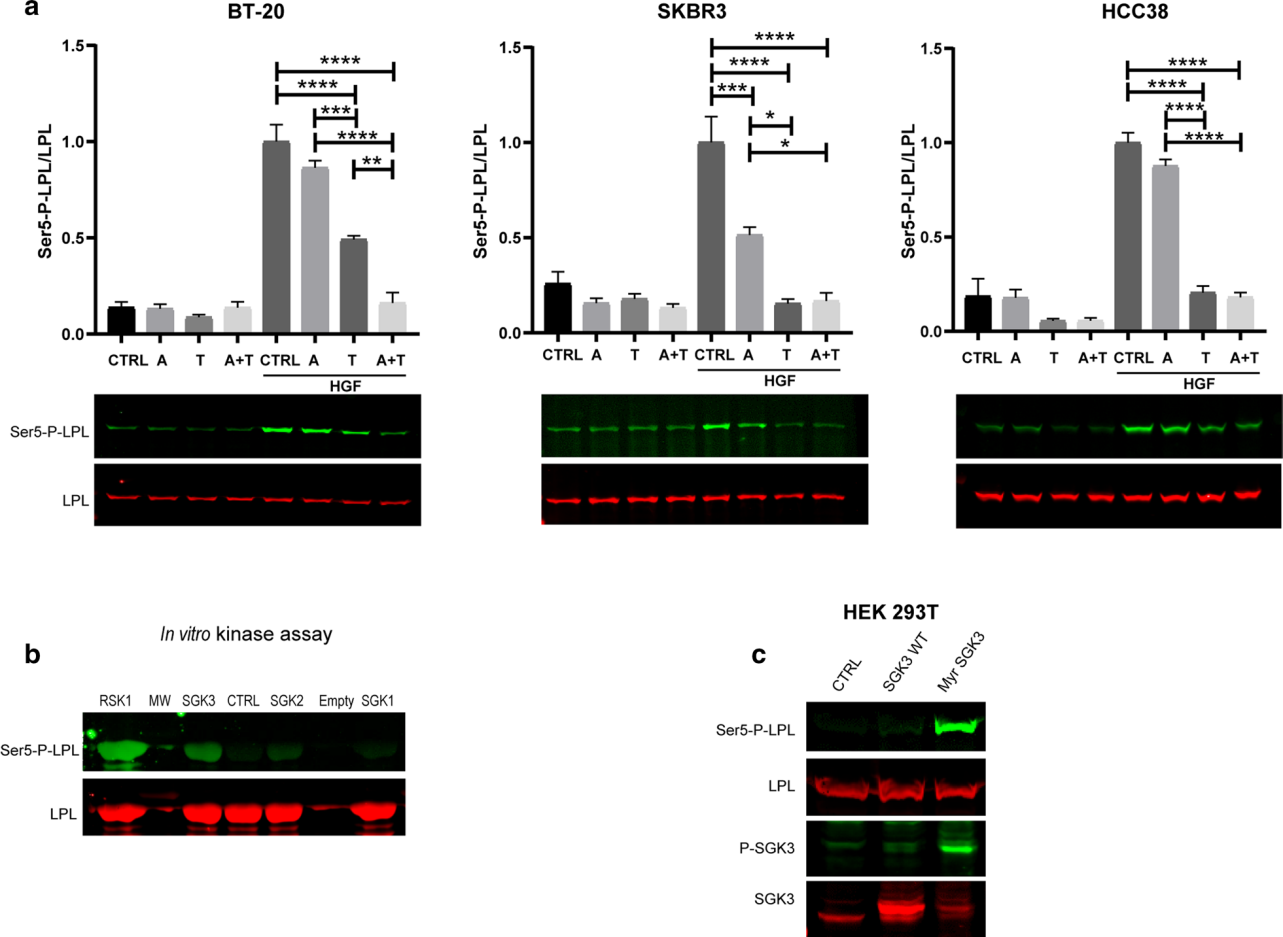
The PI3K pathway is involved in L-plastin Ser5 phosphorylation through the PI3K/SGK3 axis. **a** BT-20, SKBR3 or HCC38 cells were treated with Apitolisib (A) or Trametinib (T) or with both inhibitors (A + T), with or without subsequent HGF stimulation. Following treatment, residual L-plastin Ser5 phosphorylation and total L-plastin were determined by immunoblot analysis. The graphs show the ratio between Ser5-P-L-plastin and L-plastin. Three independent experiments were performed for each cell line. Data were scaled to the highest signal obtained (= 1) and results are expressed as means ± SEM. Statistical analysis was performed doing one-way ANOVA, relative to the control (CTRL) condition with or without HGF stimulation, respectively (*p < 0.05, **p < 0.01, ***p < 0.001, ****p < 0.0001). **b** In vitro kinase assay. A total of 10 μg recombinant full-length L-plastin was incubated with 100 ng recombinant kinase and with 50 μM ATP in a reaction volume of 25 μl. RSK1 was used as a positive control kinase and a negative control (CTRL) was performed by omitting a kinase. L-plastin Ser5 phosphorylation and total L-plastin were determined by immunoblot analysis. **c** HEK 293 T cells were co-transfected with GFP-fused L-plastinWT and FLAG-tagged SGK3 WT, activated myristoylated SGK3 (Myr SGK3) or the empty vector (Ctrl). Cell extracts were prepared 48 h after transfection and immunoblot analysis was performed to determine L-plastin Ser5 phosphorylation and total L-plastin as well as SGK3 Thr320 phosphorylation and total SGK3

In an effort to experimentally validate the involvement of the PI3K pathway in the process of L-plastin Ser5 phosphorylation, we verified the phosphorylation level of L-plastin upon pharmacological inhibition of the ERK/MAPK pathway and the PI3K pathway, either individually or combined, by treating the cells with the MEK inhibitor Trametinib and/or the dual PI3K/mTOR inhibitor Apitolisib. In BT-20 cells, the combined inhibition of both pathways consistently led to a synergistic reduction of HGF-stimulated Ser5-P-L-plastin levels (Fig. 3a). In SKBR3 cells, Apitolisib treatment reduced L-plastin Ser5 phosphorylation by 50%, whereas Trametinib treatment alone was sufficient to decrease this phosphorylation to background levels. In contrast, in HCC38 cells, Apitolisib did not display any effect, whereas Trametinib again reduced HGF-dependent L-plastin Ser5 phosphorylation to background levels. Hence, the relative contribution of the PI3K pathway to L-plastin Ser5 phosphorylation appears to depend on the cellular context.

Next, to further shed light on the possible involvement of SGKs in L-plastin activation, we performed an in vitro kinase assay assessing the ability of the three SGK isoforms SGK1, SGK2 and SGK3 to phosphorylate recombinant full-length L-plastin on residue Ser5. As shown in Fig. 3b, SGK3 was able to phosphorylate L-plastin on its residue Ser5, although to a lower extent than RSK1. SGK2 was able to induce a weak L-plastin Ser5 phosphorylation, whereas SGK1 did not exhibit such phosphorylation ability. In order to control that the three SGK isoforms were similarly active, we assessed their phosphorylation activity on the protein Mouse Double Minutes 2 MDM2, which has been described as a standard substrate of SGK1 [37]. As illustrated in Additional file 14: Figure S3, we have found that all three kinases were able to phosphorylate MDM2 on its residue Ser166, demonstrating their activity. We then examined the ability of SGK3 to phosphorylate L-plastin on its residue Ser5 in cells. To this end, we performed a comparative analysis between different SGK3 constructs by co-transfecting FLAG-tagged SGK3 WT or myristoylated SGK3 (Myr SGK3) and L-plastinWT-GFP in HEK 293T cells, which are devoid of endogenous L-plastin. As illustrated in Fig. 3c, our immunoblot analysis revealed that the myristoylated form of SGK3 was phosphorylated on residue Thr320 indicating activation of the protein. Strikingly, we consistently observed a strong L-plastin Ser5 phosphorylation only when co-expressed with the activated myristoylated SGK3 form, and not when co-expressed with the non-activated SGK3 WT, even though SGK3 WT was expressed at a higher level than myristoylated SGK3 in the cells. Altogether, our results provide evidence that L-plastin residue Ser5 can be phosphorylated in cells by SGK3, following activation of the kinase.

### L-plastin Ser5 phosphorylation modulates breast cancer cell migration and invasion

Given the important role of L-plastin in cell motility of many different cell types, we next sought to examine the functional impact of L-plastin expression and Ser5 phosphorylation level in breast cancer cells with a specific focus on cell migration and invasion. To this end, we have selected four cell lines expressing contrasting endogenous levels of this protein.

**Fig. 4:**
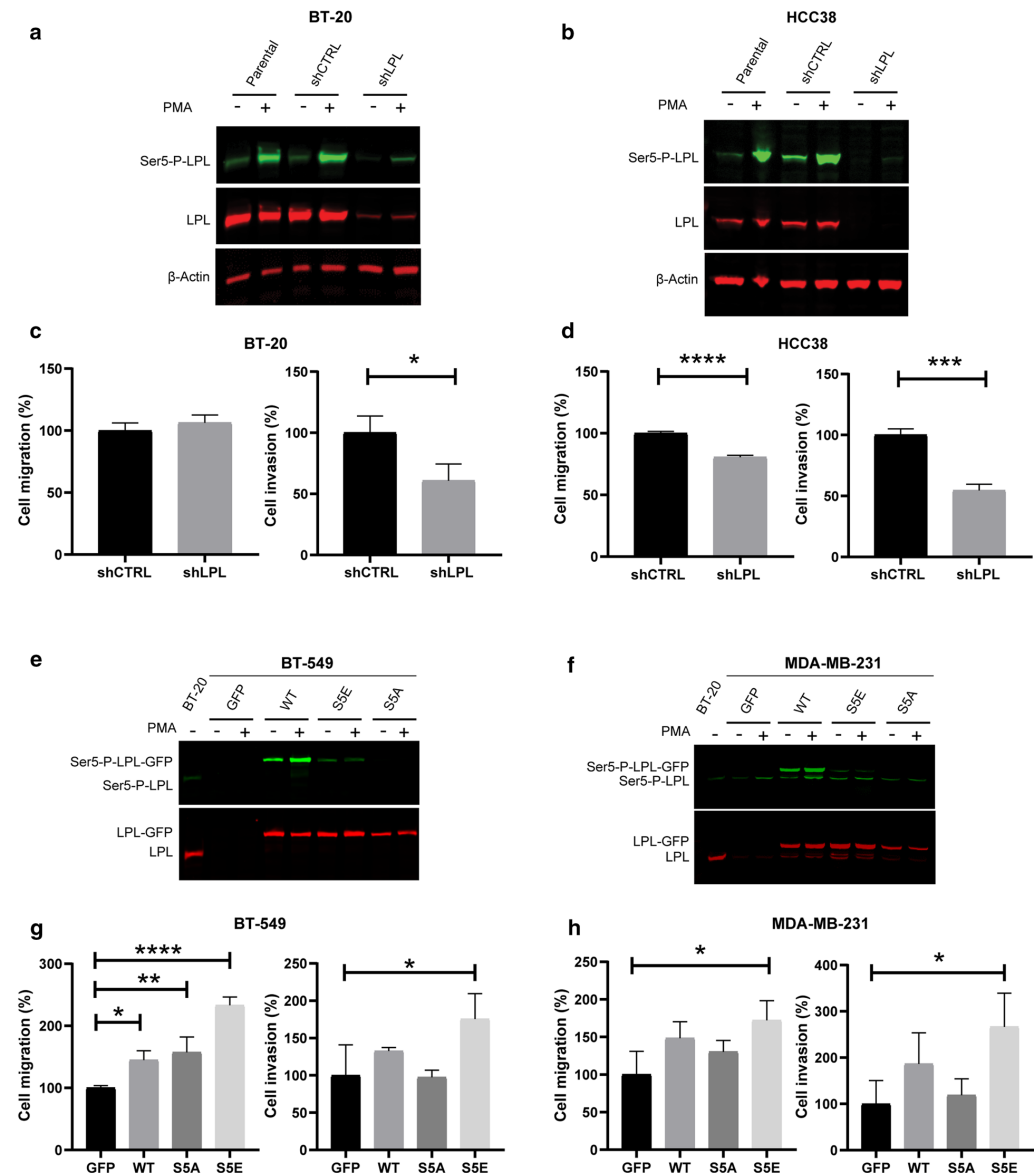
L-plastin Ser5 phosphorylation is important for migration and invasion of breast cancer cells in vitro. **a**, **b** L-plastin (red) and Ser5-P-L-plastin (green) immunoblotting of BT-20 (**a**) and HCC38 cells (**b**) transduced with shRNA control (shCTRL) and shRNA targeting L-plastin (shLPL). Cells were treated with 0.1 µM PMA for 1 h. β-actin (red) was stained as a loading control. **c**, **d** Statistical plots of transwell migration and Matrigel-coated transwell invasion assays. The number of cells which crossed the membrane was assessed after a 24 h incubation period and five fields at 20 × magnification objective were counted for each well. Three independent experiments were performed for each assay. Results are expressed as means ± SEM. Student’s t-test (*p < 0.05, ***p < 0.001, ****p < 0.0001). E) and F) L-plastin (red) and Ser5-P-L-plastin (green) immunoblotting of BT-549 (**e**) and MDA-MB-231 cells (**f**) transduced with GFP or GFP-fused L-plastinWT or the phosphorylation variants L-plastin S5E or L-plastin S5A. Cells were treated with 0.1 µM PMA for 1 h. BT-20 cell extract was loaded as a control for endogenous L-plastin expression. **g**, **h** Statistical plots of transwell migration and Matrigel-coated transwell invasion assays. The assays were performed as described under (**c**, **d**). Results are expressed as means ± SEM of three independent experiments. One way ANOVA followed by Dunnett’s multiple comparison test relative to GFP transduced cells (*p < 0.05, **p < 0.01, ****p < 0.0001)

Initially, we set out to investigate the effect of L-plastin loss-of-function in cells naturally expressing high levels of L-plastin. For that purpose, we have silenced L-plastin expression in BT-20 and HCC38 cell lines. Characterization of the transduced cells by immunoblotting revealed that the downregulation was highly efficient in both cell lines (Fig. 4a, b). Following PMA stimulation, L-plastin Ser5 phosphorylation was strongly enhanced as monitored by the stronger band corresponding to Ser5 phosphorylated L-plastin (green). Even in the cells transduced with shRNA targeting L-plastin, PMA treatment could still induce Ser5 phosphorylation of the remaining L-plastin. Next, to determine whether L-plastin silencing has an impact on cell migration and invasion capacity, we performed transwell assays with the transduced cells. We observed that the Matrigel invasion ability was significantly reduced in both cell lines whereas the transwell migration was only significantly reduced in HCC38 cells (Fig. 4c, d).

In parallel, we have neoexpressed GFP-fused L-plastin wild type (WT) or the phosphorylation variants L-plastin Ser5Ala (S5A) or L-plastin Ser5Glu (S5E) in BT-549 and MDA-MB-231 cell lines, which display no or a low level of endogenous L-plastin, respectively. Immunoblot analysis showed a band corresponding to L-plastin-GFP (red) for all the transduced cell clones, except for the one transduced with GFP alone (Fig. 4e, f). Consistently, L-plastin Ser5 phosphorylation was enhanced following PMA treatment. Of note, the anti-Ser5-P antibody also recognized weakly the phosphorylation-mimetic L-plastinS5E mutant independent of PMA treatment, whereas the phosphorylation-defective L-plastinS5A mutant was not recognized, as expected. Transwell assays showed that neoexpression of the phosphomimetic variant L-plastinS5E significantly enhances migration and invasion ability in both cell lines when compared to the GFP transduced cells (Fig. 4g, h). Importantly, this increase in invasiveness was not observed if the cells expressed the non-phosphorylatable L-plastinS5A variant. A slight increase could also be detected for L-plastinWT-GFP expressing cells, although these differences were statistically significant only for BT-549 cell migration.

Overall, these results indicate that, in addition to L-plastin expression, L-plastin Ser5 phosphorylation is required to promote breast cancer cell migration and invasion.

### L-plastin Ser5 phosphorylation promotes L-plastin recruitment to invadopodia

To further assess the role of L-plastin Ser5 phosphorylation in regulating cell migration and invasion, we performed confocal microscopy to characterize the subcellular localization of L-plastin phosphorylation variants in MDA-MB-231 cells.

**Fig. 5:**
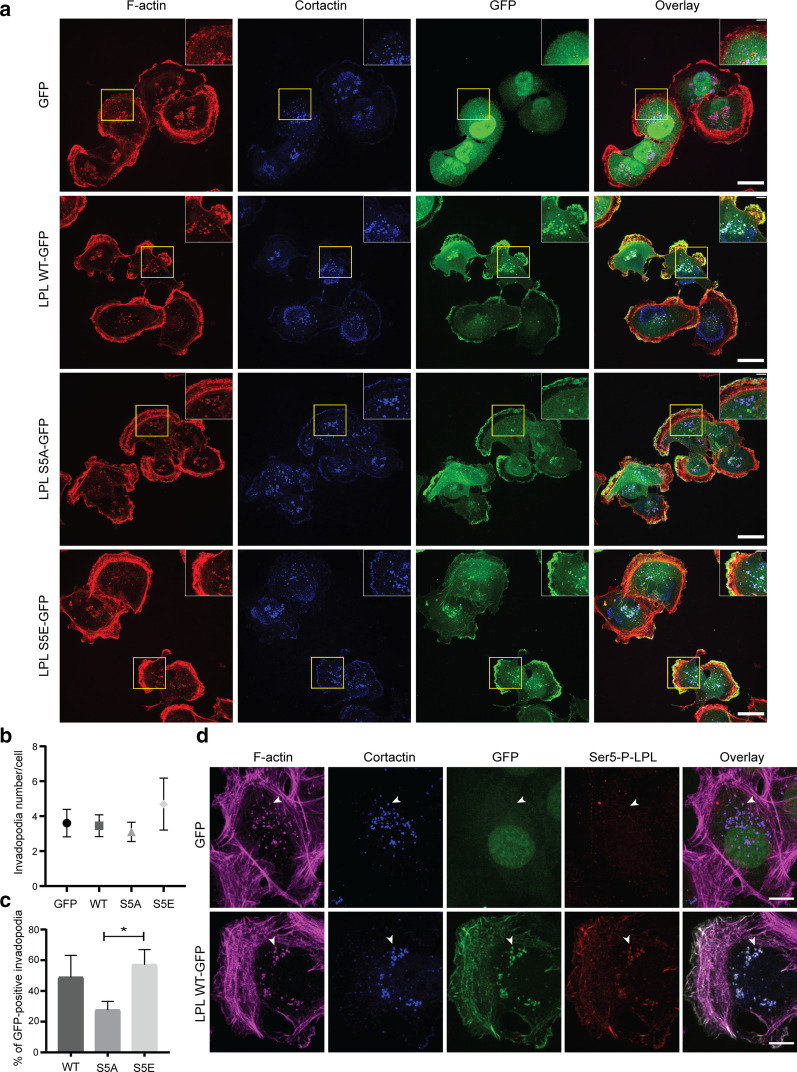
Ser5 phosphorylation enhances L-plastin recruitment to invadopodia in MDA-MB-231 cells. **a** Expression pattern of the transduced MDA-MB-231 cells expressing the different GFP-fused L-plastin constructs. Cells were plated on gelatin-coated coverslips for 24 h and stained using anti-cortactin (blue) and Alexa Fluor 594-conjugated phalloidin (red) to visualize F-actin. GFP signal was amplified using the Alexa Fluor 488-conjugated GFP booster. Scale bar: 20 µm. Areas of actin, cortactin and L-plastin co-localization are seen in the overlay as white dot-like structures (right column). The insets show a higher magnification of the boxed areas. **b** Quantification of cortactin and F-actin-containing punctae per cell was performed using single confocal slices of the ventral surface of cells. Results are expressed as means ± SEM of three independent experiments in which 60–80 cells per conditions were assessed. One way ANOVA comparing all four groups showed no significance. **c** Percentage of GFP-positive invadopodia. Results are expressed as means ± SEM of three independent experiments. One way ANOVA followed by Tukey’s multiple comparison test (*p < 0.05). **d** Co-localization of Ser5 phosphorylated L-plastin with actin and cortactin in MDA-MB-231 cells. Cells were plated onto gelatin-coated coverslips for 24 h and stained using anti-cortactin (blue) and anti-Ser5-P-L-plastin (red) antibodies, followed by Alexa Fluor 633-conjugated phalloidin (magenta) to stain F-actin. Arrowheads point to areas of co-localization of proteins, which are seen in the overlay as white dot-like structures (right column). Scale bar: 10 µm

Consistently, we observed that neoexpressed L-plastin was localized in actin-rich migratory structures and colocalized with actin and cortactin in invadopodia, with cortactin being a widely used marker of invadopodia. Invadopodia were identified as cortactin- and F-actin-containing punctae (Fig. 5a). To investigate if L-plastin regulates invadopodia formation, the number of invadopodia per cell was determined. We observed a slight increase in invadopodia density in cells expressing the phosphomimetic L-plastinS5E variant when compared to the other conditions; however, this difference was not significant (Fig. 5b). In addition, we assessed the ability of L-plastin variants to be recruited to invadopodia. Remarkably, the quantification of GFP positive invadopodia showed that the non-phosphorylatable L-plastinS5A invadopodia localization was around two-fold lower as compared to L-plastinWT or the phosphomimetic L-plastinS5E, suggesting that L-plastin Ser5 phosphorylation is critical for L-plastin recruitment to invadopodia (Fig. 5c). To strengthen this finding, we investigated the intracellular localization of Ser5 phosphorylated L-plastin using the anti-Ser5-P antibody. This approach revealed that the L-plastin recruited to invadopodia is essentially the phosphorylated form (Fig. 5d). Altogether, our results indicate that L-plastin expression does not affect invadopodia formation, but Ser5 phosphorylation facilitates L-plastin recruitment to these structures.

### L-plastin Ser5 phosphorylation does not enhance L-plastin/cortactin interaction

**Fig. 6:**
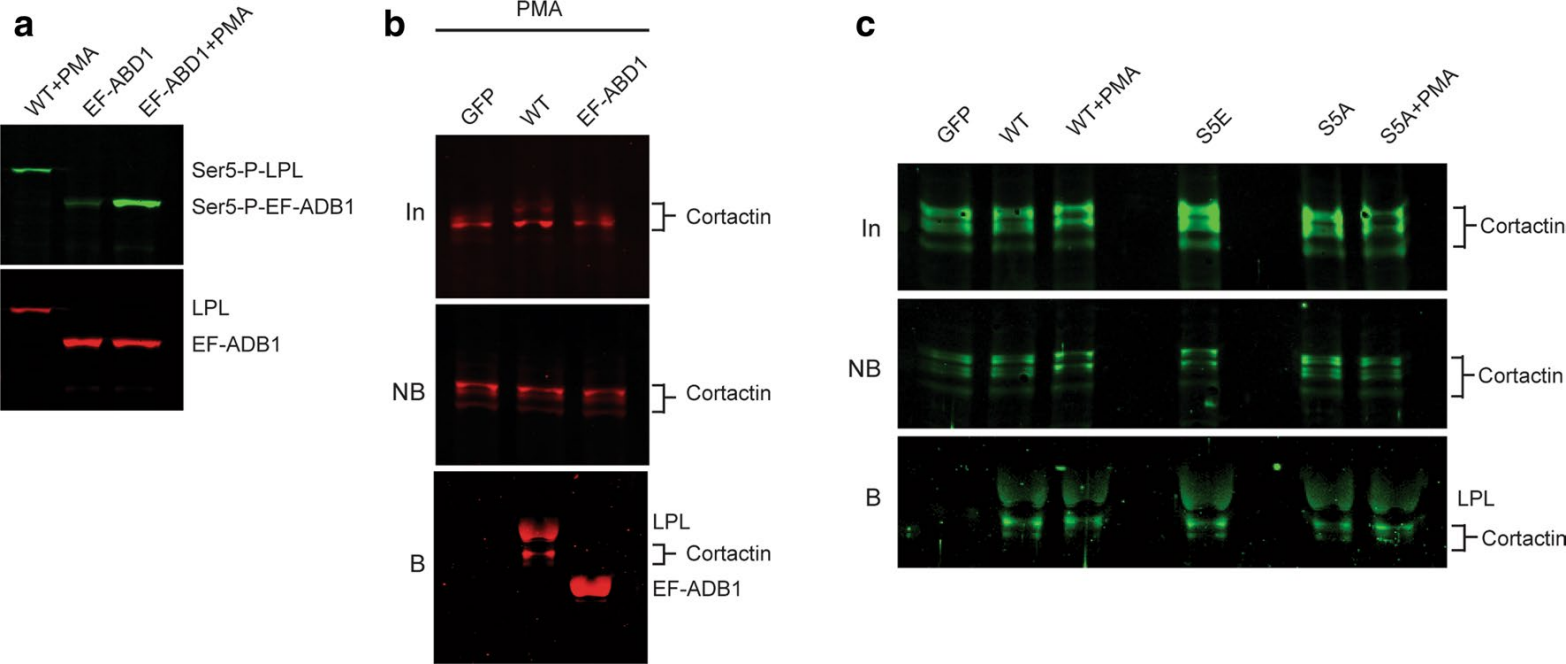
L-plastin Ser5 phosphorylation is not required for L-plastin/cortactin interaction. **a** L-plastinEF-ABD1 is phosphorylated upon PMA stimulation. HEK 293T cells were transfected with L-plastinWT-GFP or L-plastinEF-ABD1-GFP. Cells were treated with or without 0.1 μM PMA for 1 h and whole-cell lysates were submitted to L-plastin (red) and Ser5-P-L-plastin (green) immunoblotting. **b** Co-immunoprecipitation of cortactin with L-plastinWT in HEK 293T cells. Cells were transfected with GFP, L-plastinWT-GFP or L-plastinEF-ABD1-GFP and treated with 0.1 μM PMA for 1 h. Following cell lysis, protein extracts were subjected to immunoprecipitation with GFP-nanotrap. Aliquots of input [In], non-bound [NB], and bound [B] fractions were separated by SDS-PAGE and proteins were visualized by immunoblotting using an anti-cortactin antibody (#05-180). In the bound fraction, an intense unspecific signal is visible that corresponds to the important amount of precipitated L-plastinWT-GFP or L-plastinEF-ABD1-GFP. C) Co-immunoprecipitation of cortactin with L-plastinWT and L-plastin phosphovariants. Cells were transfected with GFP, L-plastinWT-GFP, L-plastinS5E-GFP and L-plastinS5A-GFP and treated with or without 0.1 μM PMA for 1 h. Protein extracts were subjected to immunoprecipitation with GFP-nanotrap. Aliquots of input [In], non-bound [NB], and bound [B] fractions were separated by SDS-PAGE and proteins were visualized by immunoblotting using an anti-cortactin antibody (#sc-11408). In the bound fraction, an intense unspecific signal is visible that corresponds to the important amount of precipitated L-plastin-GFP variants

We have previously shown that the invadopodia marker cortactin efficiently co-precipitated with L-plastinWT extracted from PMA-treated MCF7 cells [12]. Given the regulatory role of cortactin in invadopodia formation, function and assembly and knowing that cortactin acts as a scaffold protein [38], we wanted to further explore this interaction and the possible binding preference of cortactin to the Ser5 phosphorylated form of L-plastin, To this end, we performed co-immunoprecipitation experiments using GFP-nanotrap on whole-cell lysates from transfected HEK 293T cells. First, we tested if the presence of the C-terminal ABD2 (actin-binding domain 2) of L-plastin is necessary for its interaction with cortactin. To this end, we used the GFP-tagged L-plastinEF-ABD1 recombinant protein, which lacks ABD2. We certified that PMA stimulation leads to Ser5 phosphorylation of L-plastinEF-ABD1 at a similar level as compared to L-plastinWT (Fig. 6a). Cell lysates of cells transfected with GFP, L-plastinWT-GFP or L-plastinEF-ABD1-GFP treated with PMA were then submitted to GFP-nanotrap with subsequent immunoblotting (Fig. 6b). We confirmed that endogenous cortactin interacts with L-plastinWT, but the expression of L-plastinEF-ABD1 recombinant protein is not sufficient to preserve this interaction.

To investigate if Ser5 phosphorylation is essential for L-plastin/cortactin interaction, we then transfected HEK 293T cells with the GFP-tagged L-plastin phosphorylation variants (Fig. 6c). PMA stimulation of HEK 293T cells expressing L-plastinWT or expression of the phospho-mimetic L-plastinS5E did not enhance L-plastin/cortactin interaction. Likewise, the non-phosphorylatable L-plastinS5A mutant was able to co-precipitate cortactin with similar efficiency than L-plastinWT or L-plastinS5E. These observations show that Ser5 phosphorylation is not required for binding and does not enhance L-plastin/cortactin interaction.

### L-plastin Ser5 phosphorylation enhances ECM degradation ability

**Fig. 7:**
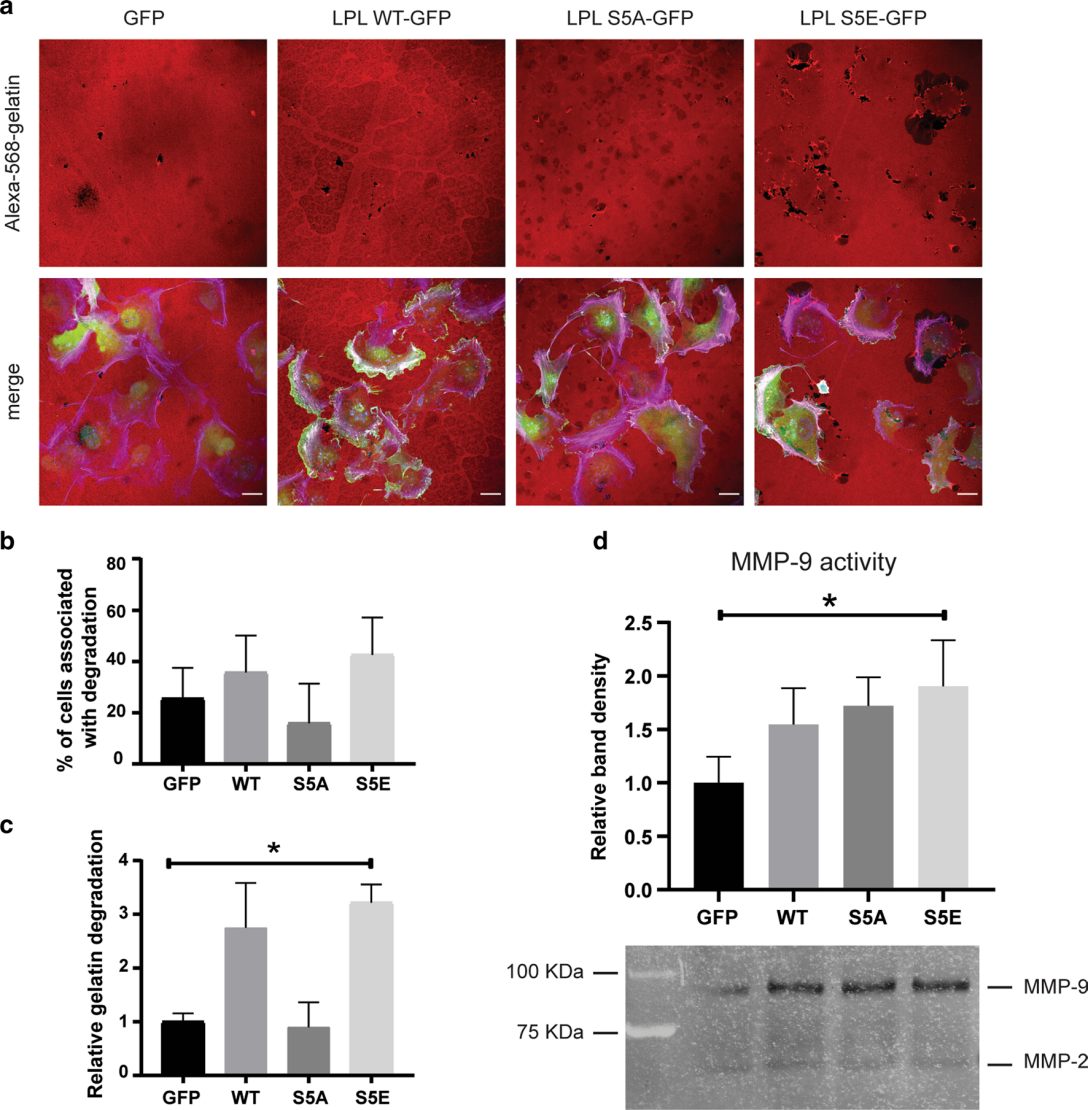
Cells expressing the phosphomimetic L-plastinS5E variant exhibit higher ability to degrade gelatin. **a** Representative images of the transduced MDA-MB-231 cells expressing the different GFP-fused L-plastin variants and degraded gelatin. Cells were plated on Alexa Fluor 568-labeled gelatin (red)-coated coverslips for 6 h and stained using Alexa Fluor 633-conjugated phalloidin (magenta) to visualize F-actin. GFP signal was amplified using the Alexa Fluor 488-conjugated GFP booster. Single confocal slices of the ventral surface of cells are shown. Areas of gelatin degradation can be identified as dark spots. Scale bar: 20 µm. **b** Quantification of cells associated with gelatin degradation. Results are expressed as means ± SEM of three independent experiments in which 100–150 cells per condition were assessed. One way ANOVA comparing all four groups showed no significance. **c** Quantification of the total degradation area normalized against total cell area. Results are expressed as means ± SEM of three independent experiments. One way ANOVA followed by Dunnett’s multiple comparison test relative to GFP transduced cells (*p < 0.05). **d** MMP activities measured by gelatin zymography. Conditioned media were collected after 24 h and equal amounts were loaded on the gel. Shown is a representative gel. The graph shows the densitometry of the MMP-9 degraded band relative to GFP control sample. Results are expressed as means ± SEM of three independent experiments. One way ANOVA followed by Dunnett’s multiple comparison test relative to GFP transduced cells (*p < 0.05)

ECM degradation activity is typically executed by mature invadopodia [39]. To monitor the impact of Ser5 phosphorylation on ECM degradation capacity, transduced MDA-MB-231 cells were plated onto fluorescent gelatin-coated coverslips (Fig. 7a). The total number of cells associated with gelatin degradation did not change significantly upon expression of L-plastin wild type or phosphorylation variants (Fig. 7b). However, an increase in gelatin degradation area was found for cells expressing L-plastinWT and the cells expressing the phosphomimetic L-plastinS5E variant were found to have the highest gelatin degradation ability as compared to GFP expressing control cells (Fig. 7c).

Greater invasive and migratory capacities are often accompanied by elevated levels of MMPs, such as MMP-2 and MMP-9 [40]. To test a possible role for L-plastin in the secretion of MMPs, we performed gelatin zymography on conditioned media of transduced MDA-MB-231 cells. Only the active forms of MMP-2 (65 kDa) and MMP-9 (82 kDa) were detected in the conditioned media. The results illustrated in Fig. 7d indicate that MMP-9, based on its gelatinase activity and apparent molecular weight, is the most prominent secreted protease in MDA-MB-231 breast cancer cells. Although all the L-plastin variants induced an increase in the activity of MMP-9, the quantification of the observed differences showed significance (~ twofold increase) only for the S5E variant when compared with GFP control samples. Collectively, our results suggest that L-plastin Ser5 phosphorylation enhances MMP-9 activity and concomitant ECM degradation.

## Discussion

Post-translational modifications (PTMs) are critical for protein function and play pivotal roles in cellular homeostasis. Aberrant PTM may contribute to pathogenesis and has been associated with numerous diseases, including cancer [41]. This implies that the exclusive analysis of genetic variations and protein expression levels may often be insufficient or even misleading [42]. In particular, protein phosphorylation displays the largest number of disease associations among PTMs and is especially relevant for breast cancer [41]. Since protein phosphorylation is frequently altered as a consequence of cancer driver gene mutations and concomitant aberrant signal transduction, phosphoproteome analysis is indispensable for the understanding of disease mechanisms and may have diagnostic and therapeutic relevance [43].

Here, we aimed at exploring the interplay between ERK/MAPK and PI3K/AKT pathways in regulating L-plastin Ser5 phosphorylation. To this end, we performed immunoblot analysis to assess the activation status of four different output nodes in four breast cancer cell lines in 20 different experimental conditions. In addition to phosphorylated L-plastin (pSer5) as an output node, we analyzed phosphorylated ERK (pTyr204) and phosphorylated AKT (pSer473) as read-outs for activated ERK/MAPK and PI3K/AKT pathways, respectively. As a fourth output node, we monitored phosphorylated c-Src (pTyr416), since activated c-Src is known to be important for tumorigenesis and cancer progression and has been previously implicated in the regulation of several signalling processes, including ERK/MAPK and PI3K/AKT pathways [44]. We have stimulated the cells with the growth factors for which they express the corresponding receptor. As inhibitors, we selected a MEK and an AKT inhibitor, to block the ERK/MAPK and PI3K/AKT pathways, respectively. Additionally, a FAK inhibitor was used, as FAK is a key molecule in invasion and metastasis and activates both ERK/MAPK and PI3K/AKT pathways [45].

Performing computational modelling based on our experimental data, we confirmed that the ERK/MAPK pathway plays a major role in L-plastin Ser5 phosphorylation. In addition, we found that the PI3K pathway likely contributes to this process via downstream SGK kinases, rather than via AKT. Our in vitro kinase assay comparing the three isoforms SGK1, SGK2 and SGK3 showed that Ser5 phosphorylation of full-lengh L-plastin is primarily mediated by the SGK3 isoform. Notably, in cells, full activation of SGK3 requires plasma or endosomal membrane localization [25, 46] mediated by the interaction of its Phox homology (PX) domain with PtdIns(3)P [47, 48]. Since myristoylation is known to target proteins to membranes [49], myristoylation of SGK3 has been shown to artificially enable full activation of SGK3 without exogenous stimulation by growth factors [22]. Here, L-plastin Ser5 phosphorylation was highly increased when co-expressed with activated myristoylated SGK3, corroborating the capacity of SGK3 to phosphorylate residue Ser5 of L-plastin in cells.

To further assess the contribution of the PI3K pathway in L-plastin Ser5 phosphorylation, we treated BT-20, HCC38 and SKBR3 cells with two drugs Apitolisib as a PI3K/mTOR inhibitor and Trametinib as a MEK inhibitor to inhibit the PI3K pathway and the ERK/MAPK pathway, respectively. The dual PI3K/mTOR inhibitor Apitolisib was chosen in order to block both PDK1, which is downstream of PI3K, and mTORC2, and thus, to inhibit the activation of SGK3, which requires phosphorylation by the two kinases [50]. In HCC38 cells, PI3K/mTOR inhibition had no major effect on HGF-triggered L-plastin Ser5 phosphorylation as compared to MEK inhibition, which strongly impaired this phosphorylation event. In contrast, in SKBR3 cells, inhibition of the PI3K/mTOR pathway reduced L-plastin Ser5 phosphorylation by 50%, and in BT-20 cells, PI3K/mTOR inhibition and MEK inhibition acted in a synergistic way to reduce this phosphorylation to background levels, suggesting that PI3K and mTOR are important for HGF-dependent L-plastin Ser5 phosphorylation in these two cell lines. Taken together, our results corroborate that the ERK/MAPK pathway is predominant for triggering L-plastin Ser5 phosphorylation. Importantly, the PI3K pathway contributes to this phosphorylation event depending on the investigated cell line. For BT-20 cells, this involvement may be explained by the presence of an activating mutation in the PI3KCA gene, which confers a gain of function to the p110α catalytic subunit of class IA PI3K and promotes PI3K-dependent tumorigenesis [51]. Oncogenic mutations in PIK3CA are found in approximately 25% of breast cancers [3]. Importantly, PI3KCA-mutant cells that lack AKT activation display a functional dependency on SGK3, which shares a consensus phosphorylation motif with AKT [52]. The mechanism linking oncogenic PI3KCA to SGK3 activation and concomitant AKT suppression involves the phosphoinositide phosphatase INPP4B, and both SGK3 and INPP4B have been suggested to have oncogenic functions [53].

Only few studies have reported so far a link between the PI3K pathway and L-plastin regulation and most of these studies have focused on the PI3K/AKT axis. In chronic lymphocytic leukemia, inhibition studies provided evidence for a role of PI3K in B-cell receptor-induced L-plastin activation through promoting Ser5 phosphorylation [54]. Moreover, in a prostate cancer study, the PI3K/AKT pathway was found to upregulate L-plastin expression levels through upregulation of the transcription activator AP4 [55]. This study showed that L-plastin is a key player in AP4-mediated prostate cancer cell migration, invasion and proliferation. Importantly, L-plastin and AP4 protein levels were also found to be upregulated in prostate cancer tissues as compared to adjacent normal tissues and correlated with lymph node metastasis. On the other hand, L-plastin has also been shown to play an upstream role in the regulation of the PI3K/AKT pathway, by promoting SDF-1α-dependent AKT Thr308 phosphorylation in human T-lymphocytes [56] or AKT Ser473 phosphorylation via regulation of the mTORC2 complex activity in a very recent hypereosinophilia study [57]. Most interestingly, immunohistochemical staining of bladder cancer tissues revealed a significant positive correlation between pAKT and L-plastin expression as well as a significant correlation between L-plastin expression and tumor histological grade, stage and growth pattern [58]. In our study, our experimental data corroborate our computational modelling predictions and indicate that, depending on the cellular context, the PI3K/SGK3 axis represents an alternative oncogenic signalling pathway involved in L-plastin activation. Knowing that this PI3K-dependent, AKT-independent signalling axis significantly contributes to cancer progression [59], it deserves close attention and SGK inhibitors might be promising therapeutic agents.

In a next step, we examined the functional impact of the L-plastin Ser5 phosphorylation event in breast cancer cells. Whereas calcium binding and oxidation of L-plastin inhibit the actin-bundling activity of L-plastin [60–63], Ser5 phosphorylation is known to promote its targeting to actin-rich structures and to increase its F-actin bundling activity in vitro and in cells [11, 12]. L-plastin phosphorylation on residues Ser5 and Ser7 has been linked to bone resorption activity via nascent sealing zone and sealing ring formation in cultured osteoclasts and in mice [64, 65]. In T-cells, L-plastin Ser5 phosphorylation is important for immunological synapse maturation and stability and, thus, for proper T-cell activation [66, 67] and, in podocytes, this phosphorylation event promotes filopodia formation [68]. Most importantly, previous results obtained in cancer cells point to the importance of this phosphorylation event for in vitro invasion and in vivo metastasis formation of melanoma cells [69, 70]. Consistent with these findings, we demonstrate here that L-plastin Ser5 phosphorylation strongly promotes cell migration and invasion capacities in a breast cancer cell model.

The process of tumor cell invasion and metastasis is associated with the assembly of invadopodia, which are F-actin-rich protrusive structures capable of degrading the ECM. These structures are characterized by the presence of the core proteins cortactin and Tks5. Moreover, they concentrate proteolytic activity and constitute the point of convergence of a plethora of signalling pathways [71]. Although, a dendritic actin network mediated by the Arp2/3 complex is known to be essential for invadopodia formation, there is increasing evidence that linear, bundled F-actin is also inherent to invadopodia [72, 73]. In addition to other actin-bundling proteins including fascin and α-actinin, a recent report has added L-plastin to the list of actin-bundling proteins found in invadopodia [19]. This study provided a model where L-plastin contributes to invadopodia extension, while protrusive force is guaranteed by fascin, which in turn, confers structural rigidity and stability [19]. In line, our results show that L-plastin ectopic expression does not affect invadopodia density in MDA-MB-231 cells, suggesting that L-plastin is not involved in the initial stage of invadopodia formation. Importantly, we found that Ser5 phosphorylation promotes L-plastin recruitment to invadopodia and L-plastin localized in invadopodia is essentially the phosphorylated form.

Since cortactin is considered as a scaffold protein in invadopodia [38], we wanted to better characterize the interaction between cortactin and L-plastin that we have shown before [12]. Our results indicate that the presence of the L-plastin ABD2 domain is necessary for this interaction, but that Ser5 phosphorylation is not required for this interaction. Given that we have shown that Ser5 phosphorylation enhances L-plastin localization in invadopodia, we can speculate that the invadopodial recruitment of L-plastin does not solely rely on its interaction with cortactin.

Degradation of the ECM by podosomes and invadopodia occurs by localized secretion of specialized proteases [74]. However, the association between L-plastin and the regulation of matrix-degrading enzymes in these structures is largely unknown. Here we demonstrated that L-plastin Ser5 phosphorylation facilitates ECM degradation although it increases only weakly MMP-9 activity. An interesting approach to study the role of L-plastin in macrophage podosomes and cancer cell invadopodia was taken by the group of Jan Gettemans, who produced nanobodies (Nb) either inhibiting L-plastin bundling activity (Nb5) or locking L-plastin in an inactivated state (Nb9) [75]. In macrophages, the expression as well as secretion and localization of the most prominent proteases MMP-2, MMP-9 and MMP-14, were found to be unaffected by both nanobodies [76]. Instead, the nanobodies induced podosome instability by affecting the cyclic turnover of actin in the podosomes and, thereby, decreased podosome lifespan. The authors concluded that defective ECM degradation observed in the nanobody-expressing cells was most likely associated with structural malformation of the podosomes [76]. Similarly, in PC-3 prostate cancer cells, the use of the L-plastin-specific nanobodies led to reduced degradation capacity of the cells [19]. Again, the bundling-inhibitor Nb5 had no effect on MMP-9 secretion and activity, confirming that Nb5-mediated reduction of degradation is not dependent on MMP-9 secretion and activity. More recently, Balta and collaborators highlighted the existence of a link between L-plastin expression, total MMP activity and MMP-2 release in MV3 melanoma cells [63]. L-plastin and MMP-2 were found to localize in invadopodial extensions and to co-immunoprecipitate, indicating that L-plastin may help MMP-2 translocate to invadopodial structures. Similarly, cortactin was shown to regulate membrane trafficking to promote protease secretion for invadopodia-associated ECM degradation [77]. A more recent study revealed a role for both cortactin and fascin in the release of extracellular vesicles containing MMPs [78]. Notably, the involvement of fascin was not linked to its actin-bundling activity, but rather to its function in microtubule-regulation and endosomal trafficking. According to our results, L-plastin expression in MDA-MB-231 cells slightly increases MMP-9 activity and has no influence on MMP-2. Despite the weakness of its influence on MMPs, we could demonstrate that L-plastin Ser5 phosphorylation strongly increases gelatin degradation capacity. Given that L-plastin Ser5 phosphorylation also increases its actin-bundling activity [11], our results suggest that enhanced degradation capacity of Ser5 phosphorylated L-plastin as compared to non-phosphorylated L-plastin is mainly due to enhanced bundling activity and only minimally due to enhanced MMP-9 activity.

Activation of invasion is a hallmark of metastatic cancers and understanding the cellular machinery that underlies invasion is critical for the establishment of novel predictive biomarkers and therapeutic targets. In this study, we have found that L-plastin Ser5 phosphorylation promotes breast cancer cell invasion, L-plastin recruitment to invasive structures and degradation of the ECM. Furthermore, we have established an involvement of the ERK/MAPK and PI3K pathways in promoting L-plastin Ser5 phosphorylation in breast cancer cell lines. In a next step, we wanted to confirm these results in tissues by analyzing the existence of potential correlations between L-plastin and ERK/MAPK or PI3K pathways in breast cancer tissue microarrays (TMAs). Surprisingly, we could not detect L-plastin in the carcinoma cells of the analyzed TMAs when performing immunohistochemistry (IHC). As L-plastin expression and Ser5 phosphorylation were detectable in infiltrated leukocytes, epitopes seem to have been preserved during sampling. This result is surprising, because L-plastin expression in breast cancer tissues has been shown before by IHC [79, 80]. Other studies have also detected an expression of L-plastin in breast cancer samples, but their methods did not take into account the heterogeneity of cancer tissues [81–83]. Whether L-plastin expression in the carcinoma cells of our breast cancer TMAs was below threshold for detection in IHC remains to be established.

## Conclusions

**Fig. 8:**
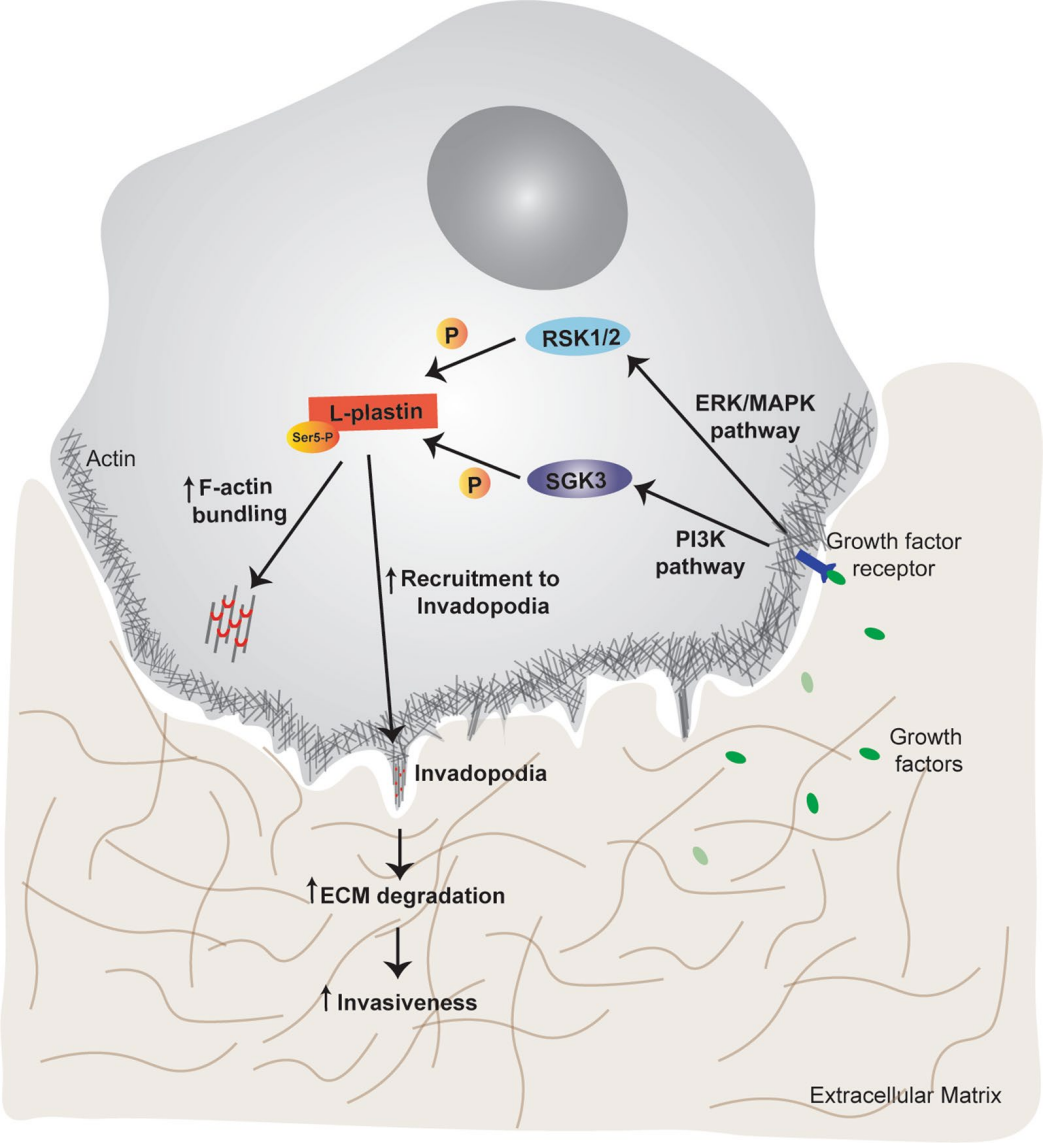
L-plastin Ser5 phosphorylation cascade in cancer cells. The ERK/MAPK and PI3K pathways are frequently dysregulated in cancer. Upon activation of these signalling pathways, their downstream effector kinases RSK1/2 and SGK3, respectively, are able to phosphorylate L-plastin on its residue Ser5. This phosphorylation leads to increased L-plastin bundling activity as well as enhanced recruitment to invadopodia and ECM degradation, promoting the invasiveness of the cancer cell

Given the extensive crosstalk between the ERK/MAPK and PI3K pathways, blocking of one of the two pathways in anti-tumor therapy might be counteracted through activation of the other pathway. Therefore, combined therapy approaches concurrently blocking both pathways are expected to have more efficient anti-tumor activities. However, complete blocking of both signalling pathways might result in significant toxicity for non-transformed cells [5]. Taking all this into consideration, one can speculate that blocking a subset of downstream functions of both pathways might be an efficient approach. As illustrated in Fig. 8, our study has provided evidence that L-plastin is a downstream molecule of both ERK/MAPK and PI3K/SGK signalling pathways and, hence, represents a potential target for such an approach. Since L-plastin Ser5 phosphorylation promotes the recruitment of L-plastin to invadopodia, ECM degradation and invasion/migration of breast cancer cells, blocking this phosphorylation event might be an interesting alternative to reduce breast cancer cell invasiveness.

## Supplementary Information

**Additional file 1: Figure S1.** Full-length blots corresponding to the immunoblots presented in Figures 1B, 1C, 3A, 3B, 3C, 4A, 4B, 4E, 4F, 6A, 6B and 6C.

**Additional file 2: Figure S4.** Driver script for the modeling of cell signalling with Dynamic Bayesian Networks. The file contains the Matlab commands to reproduce the analysis with the FALCON toolbox.

**Additional file 3: Figure S5.** Matlab workspace containing the raw analysis results.

**Additional file 4: Figure S6.** Network definition for the post-regularization model.

**Additional file 5: Figure S7.** Summary spreadsheet containing the optimal parameter values obtained by the FALCON analysis.

**Additional file 6: Figure S8.** Summary spreadsheet containing the node values for different network states under the optimal parametrization.

**Additional file 7: Figure S9.** Prior Knowledge network definition. The list of interactions specifies the topology of the signalling network used for the FALCON analysis.

**Additional file 8: Figure S10.** Constraints for the edge regularization. This empty file is used during the analysis to inform the algorithm that no prior information is used for constraining the regularization of the network model.

**Additional file 9: Figure S11.** Normalized activities for the different phosphoproteins and experimental conditions for the BT20 cell line.

**Additional file 10: Figure S12.** Normalized activities for the different phosphoproteins and experimental conditions for the HCC38 cell line.

**Additional file 11: Figure S13.** Normalized activities for the different phosphoproteins and experimental conditions for the MCF7 cell line.

**Additional file 12: Figure S14.** Normalized activities for the different phosphoproteins and experimental conditions for the SKBR3 cell line.

**Additional file 13: Figure S2.** The graphs show the ratio between the intensities obtained for phosphorylated (activated) protein versus total protein. Each ratio was then normalized to the mean of all the ratios obtained for one blot to make blots comparable by accounting for technical day-to-day variability. For representative purposes, data were scaled to the controls present on each blot and are represented as means +/- SEM of three independent experiments.

**Additional file 14: Figure S3.** A total of 2 μg recombinant full-length MDM2 was incubated with 100 ng recombinant kinase and with 50 μM ATP in a reaction volume of 25 μl. A negative control reaction (CTRL) was performed by omitting a kinase. MDM2 Ser166 phosphorylation (red) and total MDM2 (green) were determined by immunoblot analysis.

## Data Availability

All the datasets generated during this study and supporting the conclusions of this article are included within the article and its supplementary figures.

## Abbreviations

A: Apitolisib
ABD: Actin-binding domain
AKT: RAC-α serine/threonine kinase
ARP2/3: Actin-related protein 2/3 complex
ATP: Adenosine triphosphate
BIC: Bayesian information criterion
BID: BiD1870, RSK inhibitor
CTRL: Control
DAPI: 4′,6-Diamidino-2-phenylindole
E, Glu: Glutamic acid
ECM: Extracellular matrix
EDTA: Ethylenediaminetetraacetic acid
EGF: Epidermal growth factor
EGFR: Epidermal growth factor receptor
ERK: Extracellular signal regulated kinase (MAPK3)
FAK: Focal adhesion kinase
GFP: Green fluorescent protein
HEK: Human embryonic kidney
HGF: Hepatocyte growth factor
HGFR: Hepatocyte growth factor receptor
IGF: Insulin-like growth factor 1
IGF-IR: Insulin-like growth factor 1 receptor
IHC: Immunohistochemistry
INPP4B: Inositol polyphosphate-4-phosphatase type II B
kDa: Kilodalton
LPL: Total L-plastin
MAPK: Mitogen-activated protein kinase
MDM2: Mouse double minutes 2
MEK: Mitogen-activated protein kinase kinase (MAP2K)
MMP: Matrix metalloproteinase
MSE: Mean squared error
mTOR: Mammalian target of rapamycin
Nb: Nanobody
PBS: Phosphate-buffered saline
PCR: Polymerase chain reaction
PDK1: 3-Phosphoinositide-dependent protein kinase 1
PFA: Paraformaldehyde
PI3K: Phosphoinositide-3 kinase
PI3KCA: Phosphoinositide-3 kinase catalytic subunit α
PKA: Protein kinase A
PKC: Protein kinase C
PMA: Phorbol 12-myristate 13-acetate
p70S6K: P70 ribosomal protein S6 kinase
PTM: Post-translational modification
rpm: Rotation per minute
ROI: Region of interest
RSK1: P90 ribosomal protein S6 kinase α-1
RSK2: P90 ribosomal protein S6 kinase α-3
RTK: Receptor tyrosine kinase (growth factor receptor)
S, Ser: Serine
Ser5-P-LPL: Ser5 phosphorylated L-plastin
SD: Standard deviation
SDF-1α: Stromal cell-derived factor 1α
SEM: Standard error of the mean
SGK: Serum/glucocorticoid regulated kinase
shRNA: Short hairpin RNA
Src: Sarcoma
T: Trametinib
Tks5: SH3 and PX domain-containing protein 2A
TMA: Tissue microarray
WT: Wild type
