# Supplementary material for: L-plastin Ser5 phosphorylation is modulated by the PI3K/SGK pathway and promotes breast cancer cell invasiveness
Source: Cell Commun Signal. 2021 Feb 22;19:22. doi: 10.1186/s12964-021-00710-5 (PMC7898450; doi:10.1186/s12964-021-00710-5)

Figure S1

Figure 1B

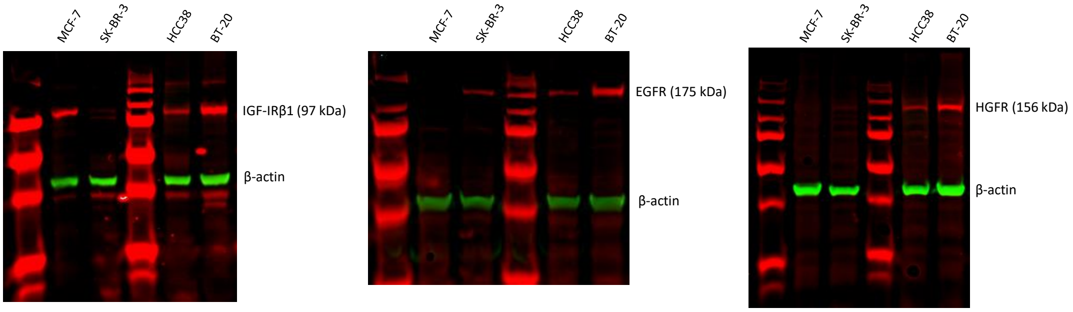

Figure 1C

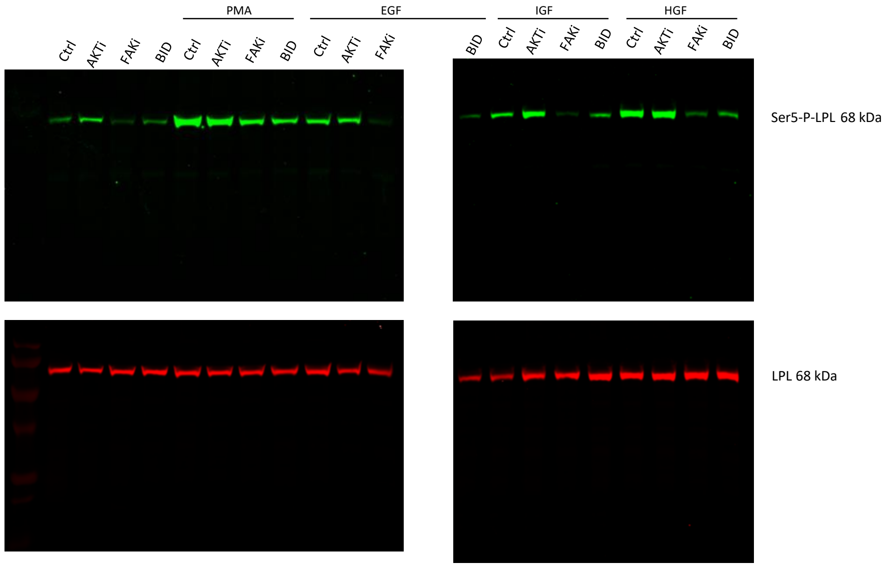

Figure 3A

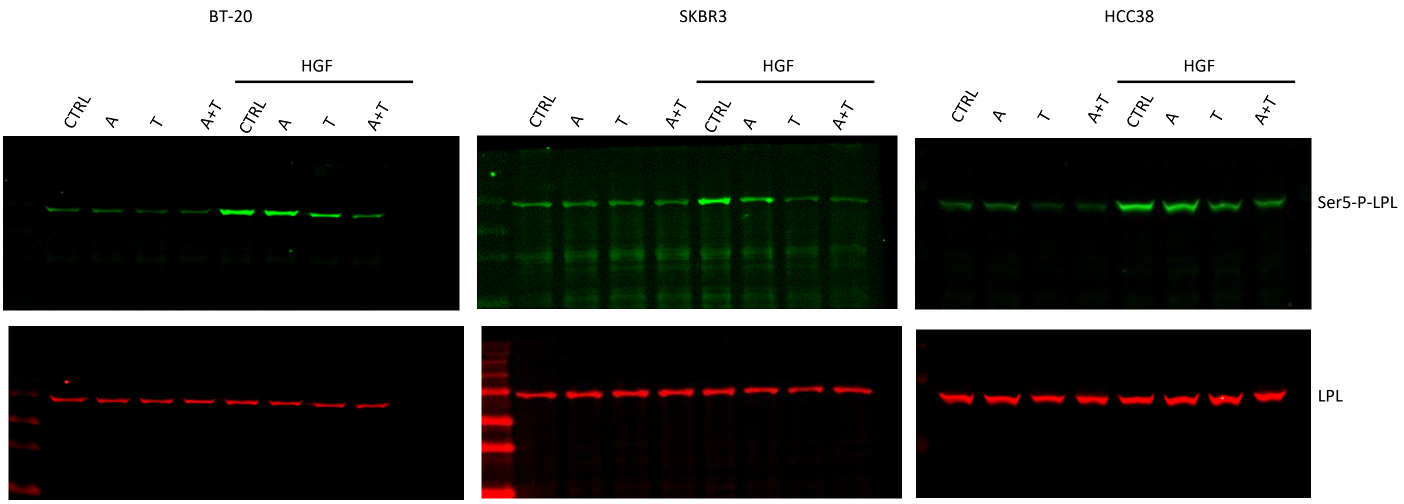

Figure 3B

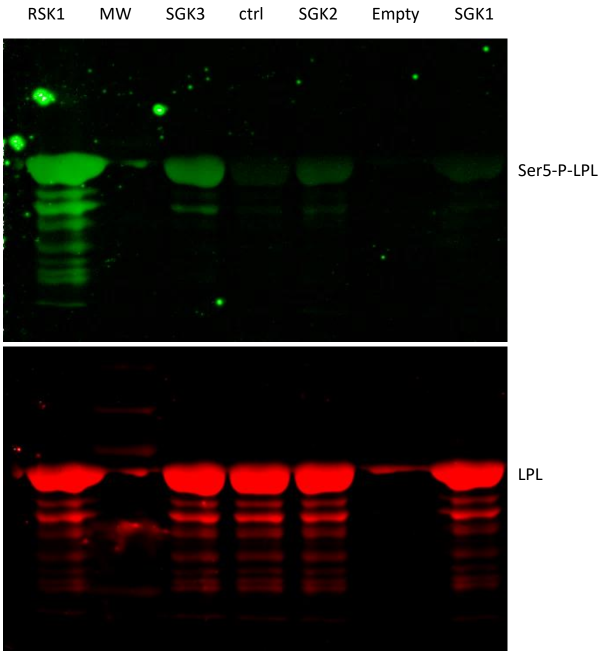

Figure 3C

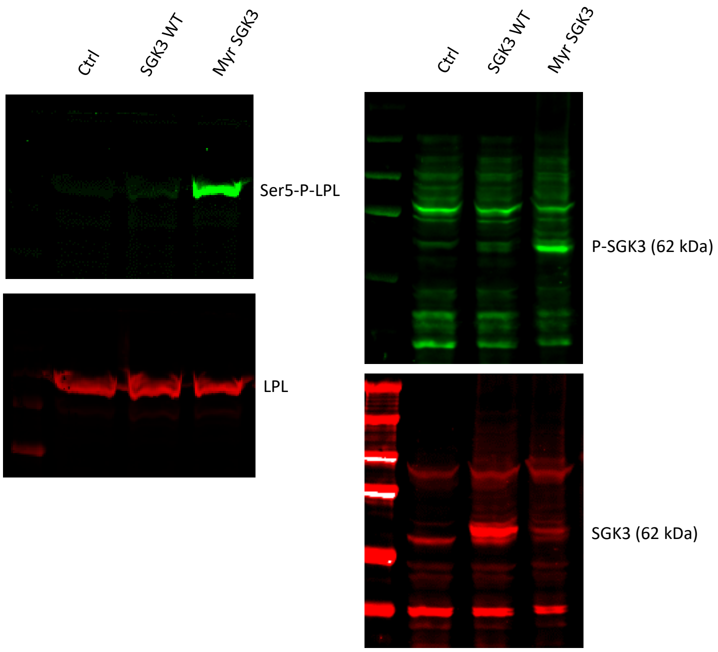

Figure 4A

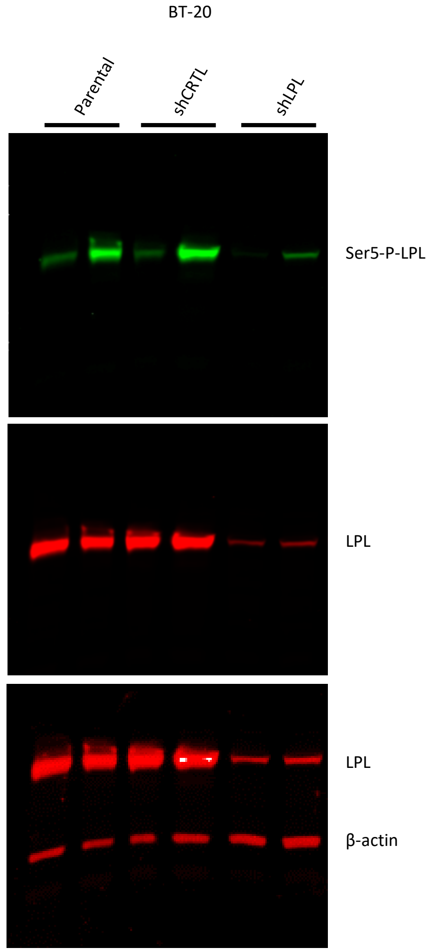

Figure 4B

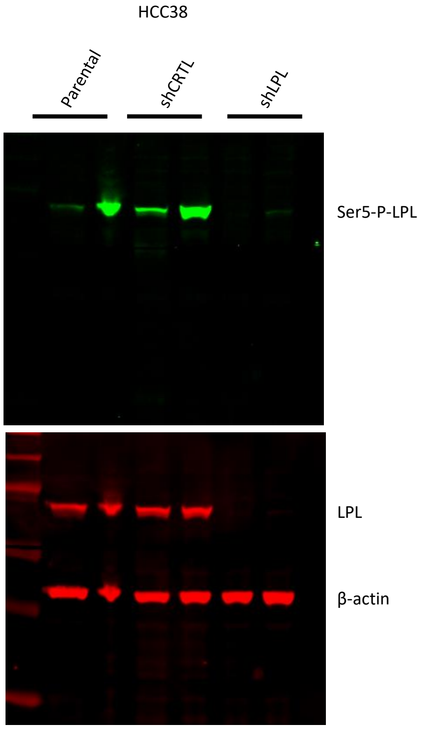

Figure 4E

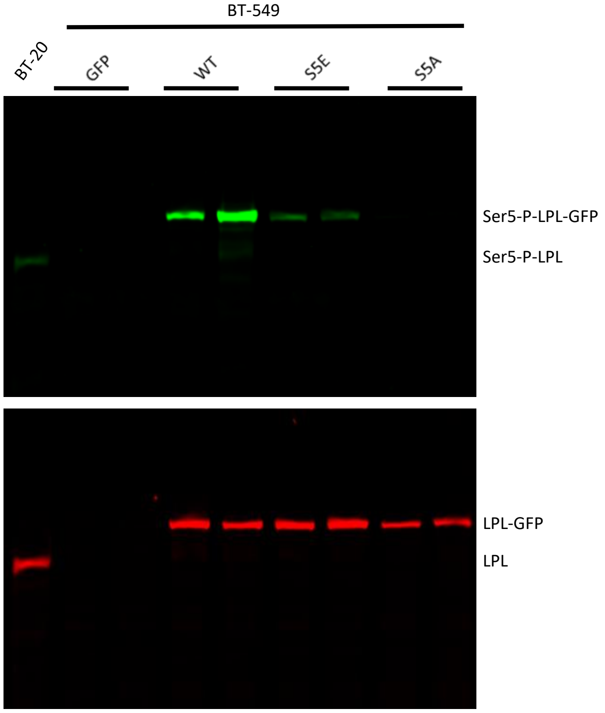

Figure 4F

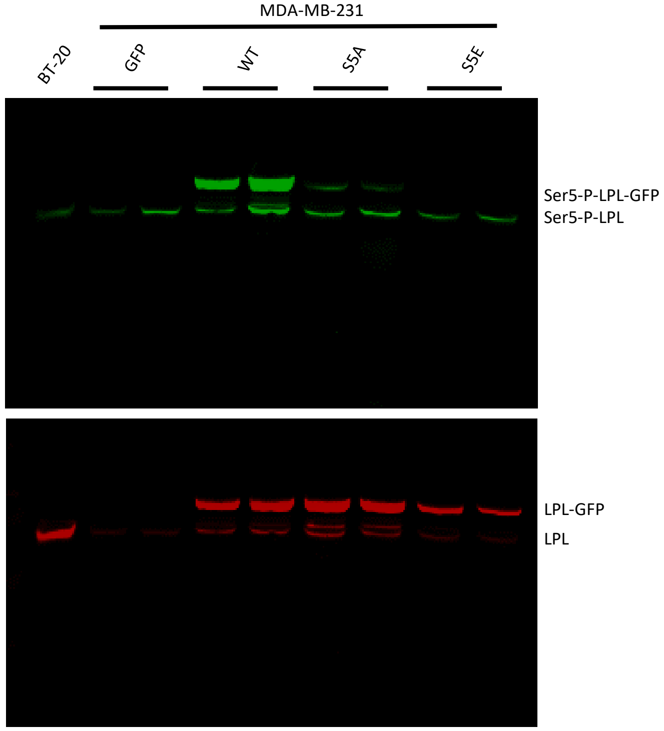

Figure 6A

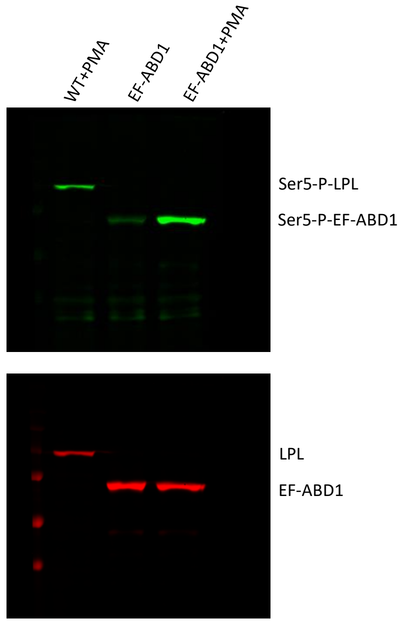

Figure 6B

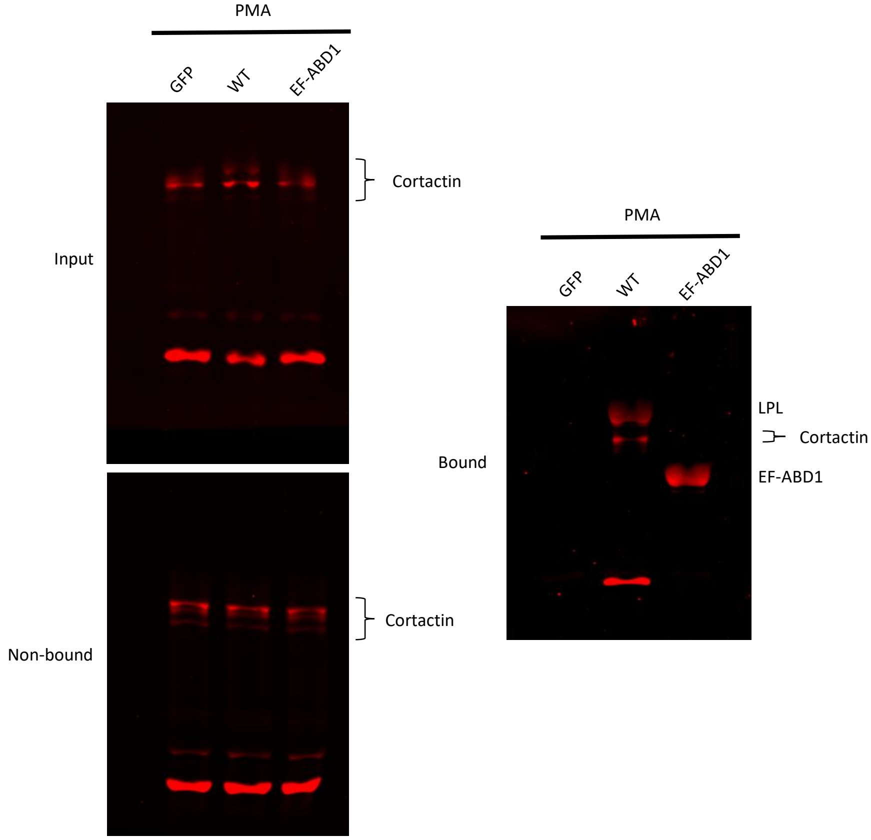

Figure 6C

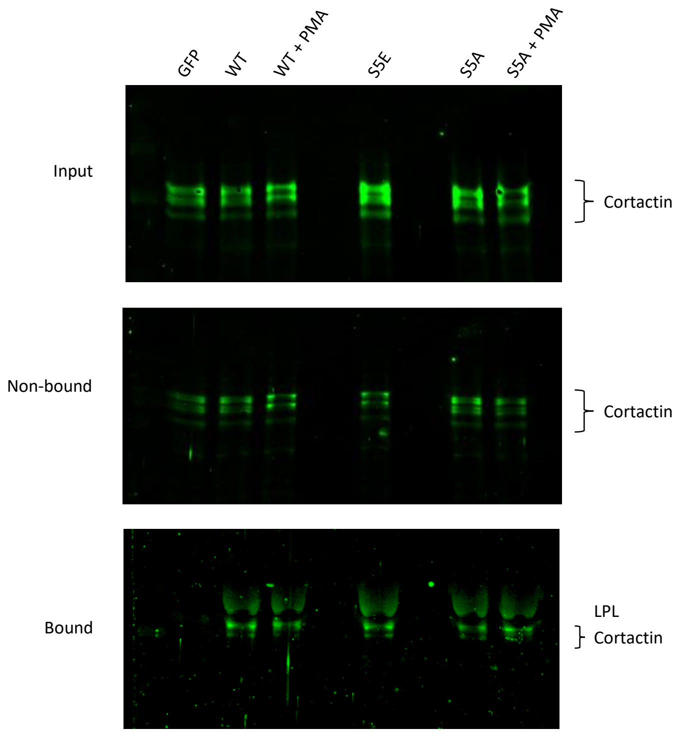

Supplement: Supplementary file 2 — Additional file 1: Figure S1. Full-length blots corresponding to the immunoblots presented in Figures 1B, 1C, 3A, 3B, 3C, 4A, 4B, 4E, 4F, 6A, 6B and 6C. [file 12964_2021_710_MOESM2_ESM.pdf]
