# Supplementary figures and images for: L-plastin Ser5 phosphorylation is modulated by the PI3K/SGK pathway and promotes breast cancer cell invasiveness
Source: Cell Commun Signal. 2021 Feb 22;19:22. doi: 10.1186/s12964-021-00710-5 (PMC7898450; doi:10.1186/s12964-021-00710-5)

# Figure S2

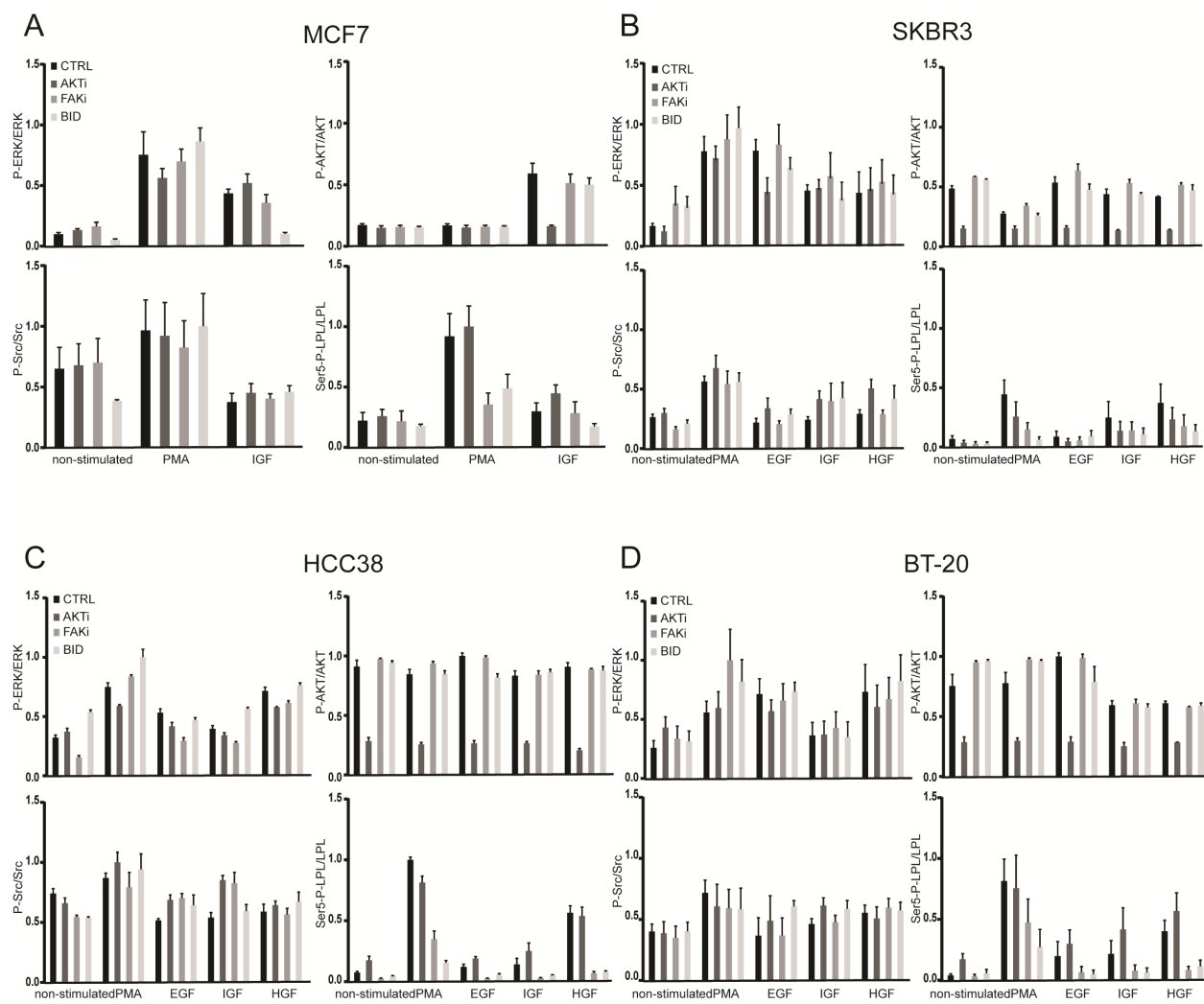

Supplement: Supplementary file 14 — Additional file 13: Figure S2. The graphs show the ratio between the intensities obtained for phosphorylated (activated) protein versus total protein. Each ratio was then normalized to the mean of all the ratios obtained for one blot to make blots comparable by accounting for technical day-to-day variability. For representative purposes, data were scaled to the controls present on each blot and are represented as means +/- SEM of three independent experiments. [file 12964_2021_710_MOESM14_ESM.pdf]

Figure S3

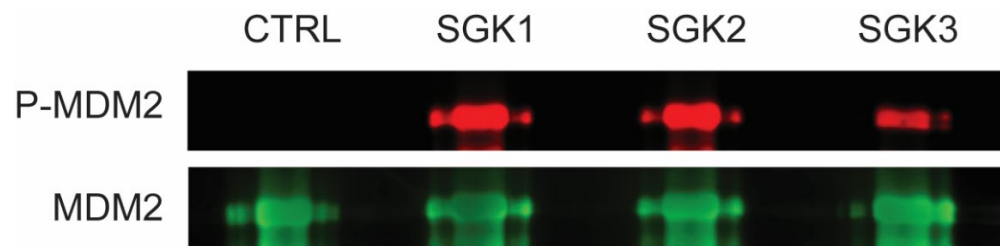

Supplement: Supplementary file 15 — Additional file 14: Figure S3. A total of 2 μg recombinant full-length MDM2 was incubated with 100 ng recombinant kinase and with 50 μM ATP in a reaction volume of 25 μl. A negative control reaction (CTRL) was performed by omitting a kinase. MDM2 Ser166 phosphorylation (red) and total MDM2 (green) were determined by immunoblot analysis. [file 12964_2021_710_MOESM15_ESM.pdf]
